# Supplementary material for: Targeting DNA2 overcomes metabolic reprogramming in multiple myeloma
Source: Nat Commun. 2024 Feb 8;15:1203. doi: 10.1038/s41467-024-45350-8 (PMC10853245; doi:10.1038/s41467-024-45350-8)

Supplementary information

Targeting DNA2 Overcomes Metabolic Reprogramming in Multiple Myeloma

Natthakan Thongon[1], Feiyang Ma[2], Natalia Baran[1], Pamela Lockyer[1], Jintan Liu[3], Christopher Jackson[1], Ashley Rose[1], Ken Furudate[1,] Bethany Wildeman[1], Matteo Marchesini[4], Valentina Marchica[5], Paola Storti[5], Giannalisa Todaro[5], Irene Ganan-Gomez[1], Vera Adema[1], Juan Jose Rodriguez-Sevilla[1], Yun Qing[6], Min Jin Ha[6], Rodrigo Fonseca[7], Caleb Stein[7], Caleb Class[8], Lin Tan[9], Sergio Attanasio[3], Guillermo Garcia-Manero[1], Nicola Giuliani[5], David Berrios Nolasco[10], Andrea Santoni[1], Claudio Cerchione[4], Carlos Bueso-Ramos[11], Marina Konopleva[1], Philip Lorenzi[9], Koichi Takahashi[1], Elisabet Manasanch[10], Gabriella Sammarelli[5], Rashmi Kanagal-Shamanna[11], Andrea Viale[3], Marta Chesi[7], and Simona Colla[1]*

[1]Department of Leukemia, The University of Texas MD Anderson Cancer Center, Houston, TX, USA.

[2]Division of Rheumatology, Department of Internal Medicine, Michigan Medicine, University of Michigan, Ann Arbor, MI, USA.

[3]Department of Genomic Medicine, The University of Texas MD Anderson Cancer Center, Houston, TX, USA.

[4]IRCCS Instituto Romagnolo per lo Studio dei Tumori (IRST) Dino Amadori, Meldola, Italy.

[5]Department of Medicine and Surgery, University of Parma, Parma, Italy.

[6]Department of Biostatistics, The University of Texas MD Anderson Cancer Center, Houston, TX, USA.

[7]Department of Medicine, Mayo Clinic, Scottsdale, AZ, USA.

[8]Department of Pharmaceutical Sciences, College of Pharmacy and Health Sciences, Butler University, Indianapolis, IN, USA.

[9]Metabolomics Core Facility, Department of Bioinformatics and Computational Biology, The University of Texas MD Anderson Cancer Center, Houston, TX, USA.

[10]Department of Lymphoma and Myeloma, The University of Texas MD Anderson Cancer Center, Houston, TX, USA.

[11]Department of Hemopathology, The University of Texas MD Anderson Cancer Center, Houston, TX, USA.

***Corresponding Author:** Simona Colla, Ph.D.

The University of Texas MD Anderson Cancer Center, Department of Leukemia

1515 Holcombe Blvd, Unit 428, Houston, TX 77030

Email: scolla@mdanderson.org ; Phone: (713) 745-7331; Fax: (713) 794-4297

Supplementary Figure 1.

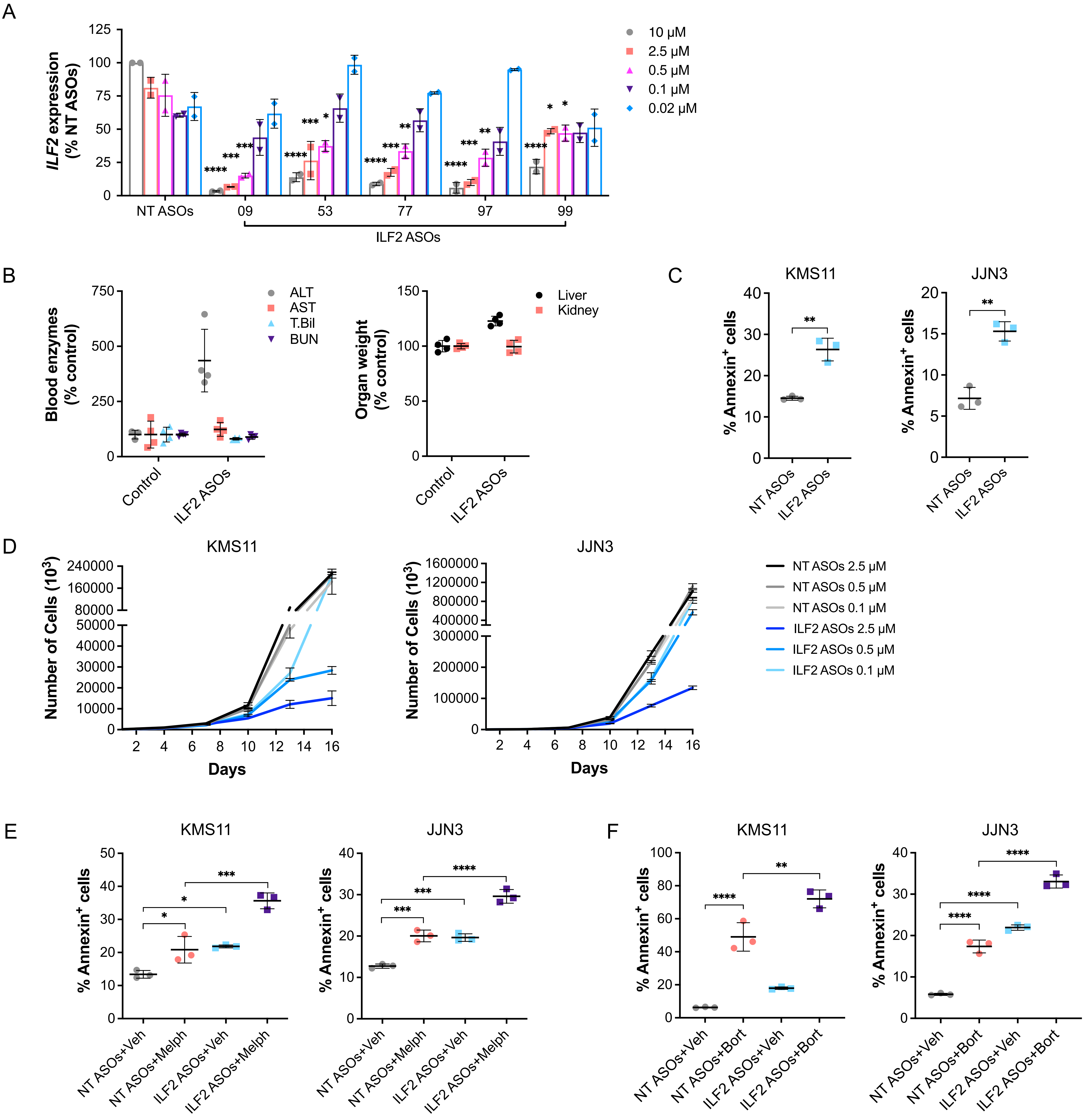

Supplementary Figure 1 (continue)

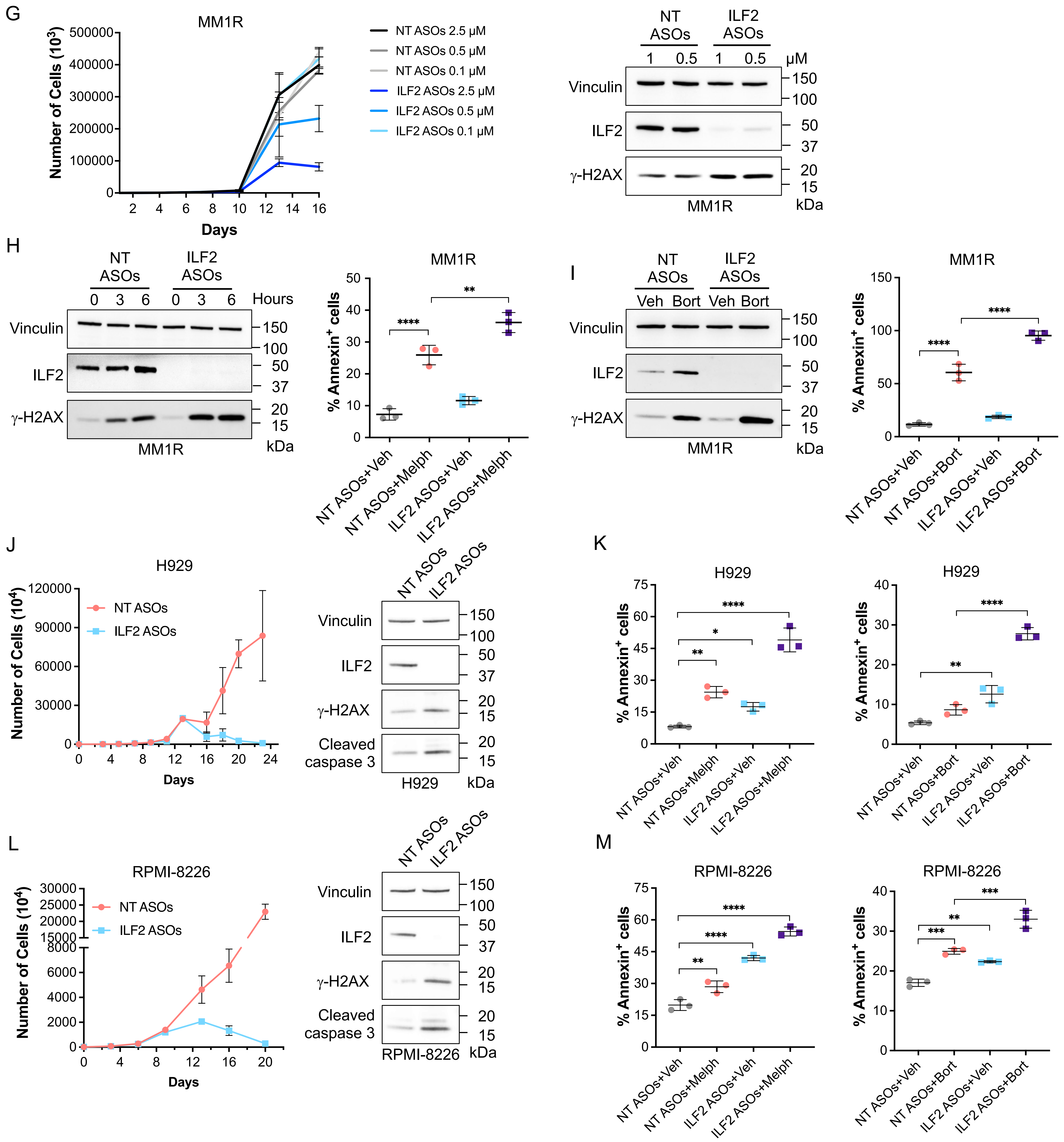

Supplementary Figure 1 (continue)

N

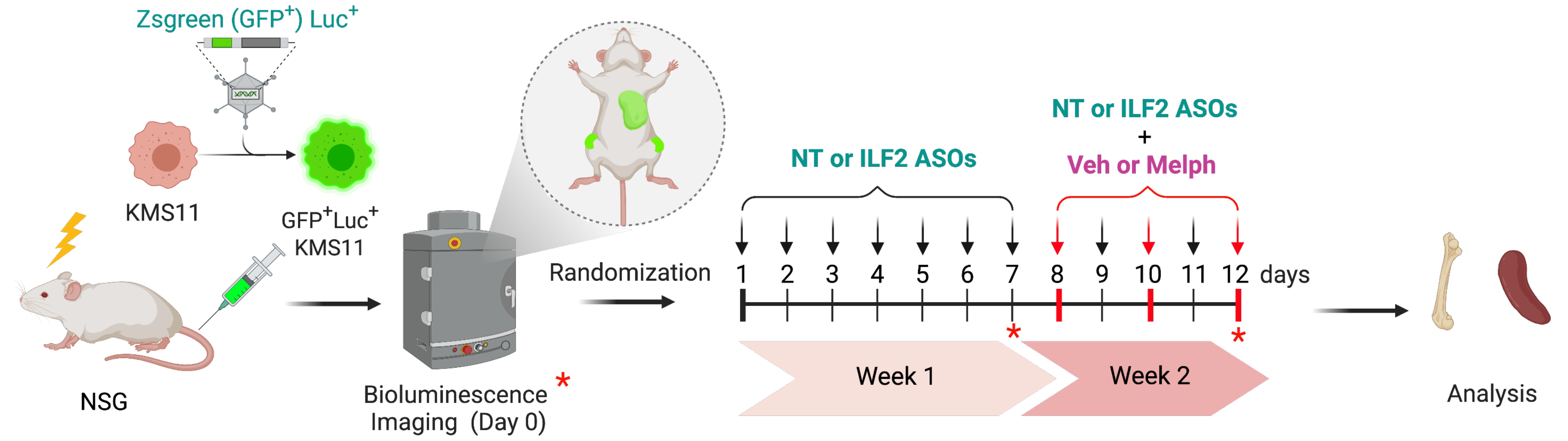

O

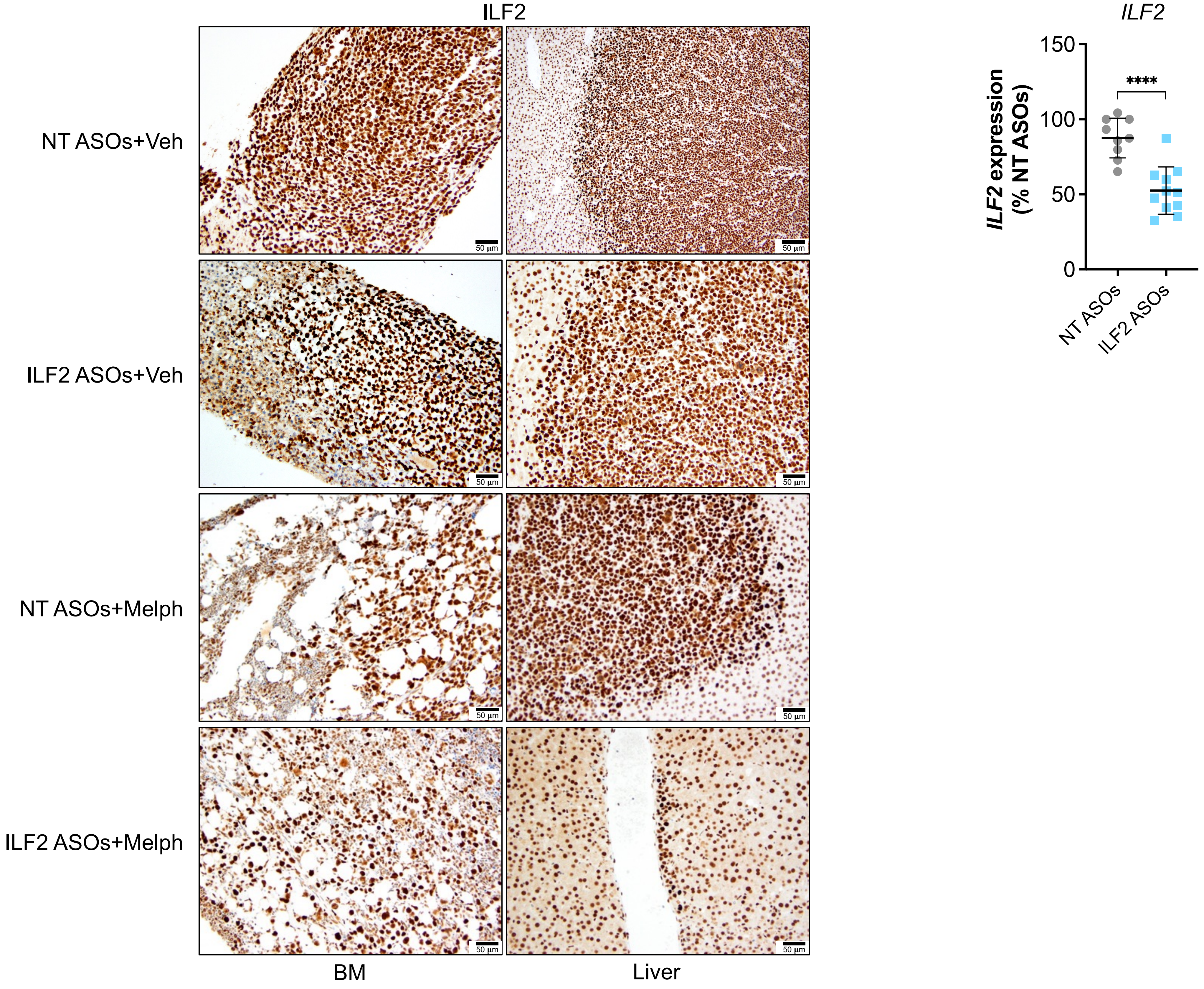

Supplementary Figure 1 (continue)

P

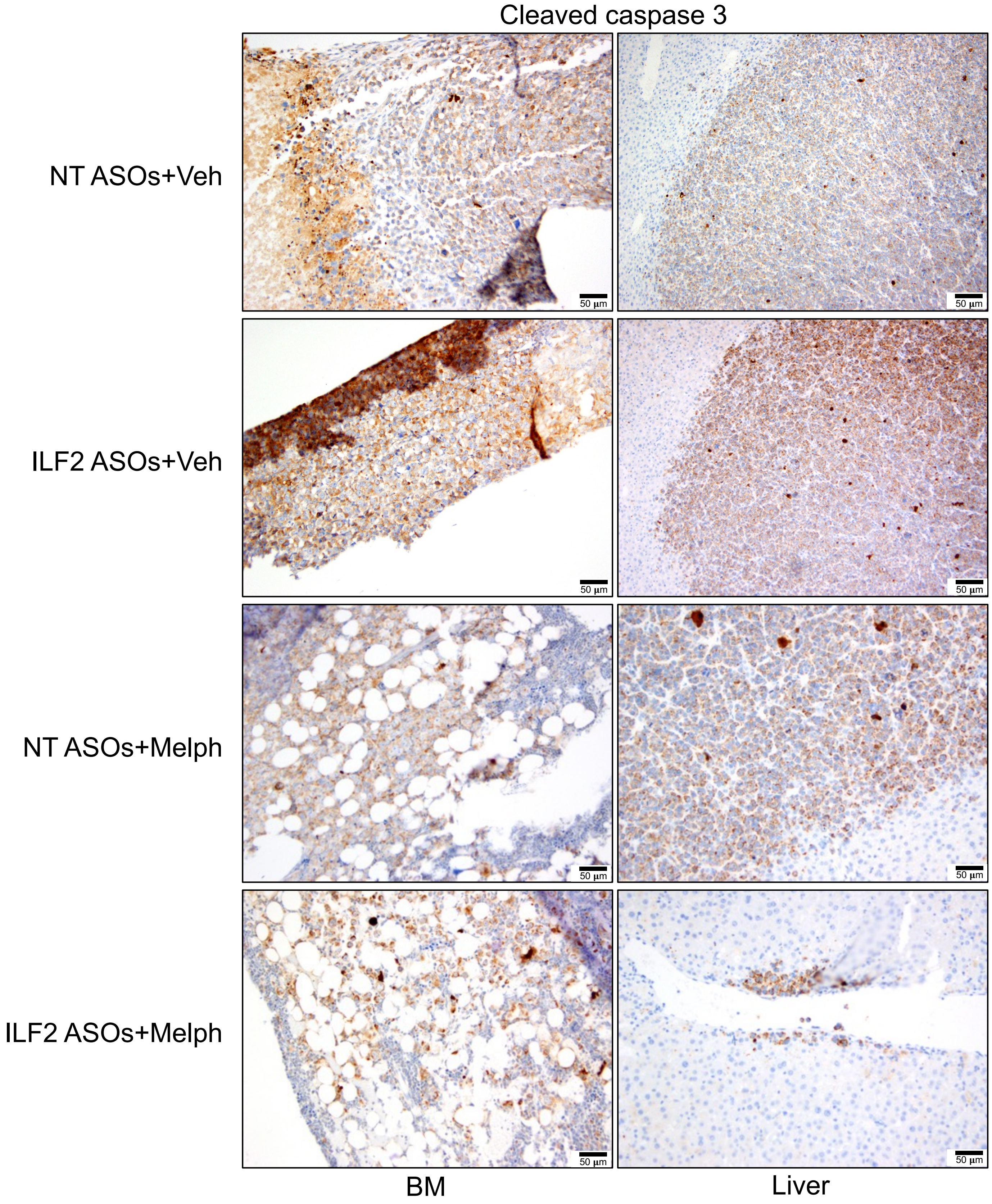

Supplementary Figure 1. ILF2-ASOs induce DNA damage activation and enhance MM cells' sensitivity to DNA-damaging agents.

(**A**) Relative *ILF2* expression in JJN3 cells treated with 5 different ILF2 ASOs at the indicated concentrations for 72 hours (the last 2 digits of the ILF2 ASOs' identification number from Supplementary Table 1 are shown). The mean ± S.D. of 2 independent experiments is shown; data are expressed as percentages of *ILF2* expression in cells treated with 10 µM NT ASOs. Statistically significant differences between ILF2 ASOs vs NT ASOs of each concentration were detected using one-way ANOVA (*****P* <0.0001; ****P* <0.001; ***P* <0.01; **P* <0.05).
(**B**) Left, levels of alanine aminotransaminase (ALT), aspartate aminotransaminase (AST), total bilirubin (T. Bil), and blood urea nitrogen (BUN) in the peripheral blood of Balb/c mice treated with phosphate-buffered saline (control; n=4) or the human ILF2 ASO 09 (n=4; 50 mg/kg delivered twice weekly by intraperitoneal injection). Right, relative weights of the liver and kidneys in each mouse.
(**C**) Frequencies of apoptotic KMS11 (left) and JJN3 (right) cells after 1 week of treatment with NT or ILF2 ASOs (0.5 µM or 1 µM, respectively). Data are presented as the mean ± S.D. from one representative experiment performed in triplicate. Statistically significant differences were detected using a 2-tailed Student t-test (***P* <0.01).
(**D**) Growth curves of KMS11 (left) or JJN3 (right) cells treated with NT or ILF2 ASOs at the indicated concentrations for 16 days. Data are presented as the mean ± S.D. from one representative experiment performed in triplicate. Live cells were counted using Trypan Blue staining.
(**E**) Frequencies of apoptotic KMS11 (left) and JJN3 (right) cells after treatment with NT or ILF2 ASOs (0.5 µM or 1 µM, respectively) for 1 week prior to receiving vehicle (Veh) or melphalan (Melph; 2 µM) for 48 hours. Data are presented as the mean ± S.D. from one representative experiment performed in triplicate. Statistically significant differences were detected using two-way ANOVA (*****P*<0.0001; ****P* <0.001; **P* <0.05).
(**F**) Frequencies of apoptotic KMS11 (left) and JJN3 (right) cells after treatment with NT or ILF2 ASOs (0.5 µM or 1 µM, respectively) for 1 week prior to receiving vehicle (Veh) or bortezomib (Bort; 5 nM) for 48 hours. Data are presented as the mean ± S.D. from one representative experiment performed in triplicate. Statistically significant differences were detected using two-way ANOVA (*****P* <0.0001; ***P* <0.01).
(**G**) Left, growth curves of MM1R cells treated with NT or ILF2 ASOs at the indicated concentrations for 16 days. The mean ± S.D. of duplicates from one representative experiment performed in duplicates are shown. Right, Western blot analysis of ILF2 and γH2AX in MM1R cells treated for 1 week with NT or ILF2 ASOs at the indicated concentrations. Vinculin was used as a loading control.
(**H**) Left, Western blot analysis of ILF2 and γH2AX in MM1R cells treated with NT or ILF2 ASOs (1 µM) for 1 week prior to receiving melphalan (Melph; 10 µM) for 3 or 6 hours. Vinculin was used as a loading control. Right, frequencies of apoptotic MM1R cells treated with NT or ILF2 (1 µM) ASOs for 1 week prior to receiving vehicle (Veh) or Melph (2 µM) for 48 hours. Data are presented as the mean ± S.D. from one representative experiment performed in triplicate. Statistically significant differences were detected using two-way ANOVA (*****P* <0.0001; ***P* <0.01).
(**I**) Left, Western blot analysis of ILF2 and γH2AX in MM1R cells treated with NT or ILF2 ASOs (1 µM) for 1 week prior to receiving vehicle (Veh) or bortezomib (Bort; 5 nM) for 48 hours. Vinculin was used as a loading control. Right, frequencies of apoptotic MM1R cells treated with NT or ILF2 ASOs (1 µM) for 1 week prior to receiving Veh or Bort (5 nM) for 48 hours. Data are presented as the mean ± S.D. from one representative experiment performed in triplicate. Statistically significant differences were detected using two-way ANOVA (*****P* <0.0001).
(**J**) Left, growth curves of H929 cells treated with NT or ILF2 ASOs (2 µM) for 23 days. The mean ± S.D. of duplicates from one representative experiment performed in duplicates are shown. Right, Western blot analysis of ILF2, γH2AX, and cleaved caspase 3 in H929 cells treated for 7 days with NT or ILF2 ASOs (2 µM). Vinculin was used as a loading control.
(**K**) Frequencies of apoptotic H929 cells treated with NT or ILF2 ASOs (2 µM) for 1 week prior to exposure to vehicle (Veh) or Melph (2 µM) for 48 hours (left) or vehicle (Veh) or bortezomib (Bort; 5 nM) for 48 hours; right. Data are the mean ± S.D. from one representative experiment performed in triplicates. Statistically significant differences were detected using two-way ANOVA (*****P* <0.0001; ***P* <0.01; **P* <0.05).
(**L**) Left, growth curves of RPM1-8226 cells treated with NT or ILF2 ASOs (1 µM) for 20 days. The mean ± S.D. of duplicates from one representative experiment performed in duplicates are shown. Right, Western blot analysis of ILF2, γH2AX, and cleaved caspase 3 in RPMI-8226 cells treated for 7 days with NT or ILF2 ASOs (1 µM). Vinculin was used as a loading control.
(**M**) Frequencies of apoptotic RPMI-8226 cells treated with NT or ILF2 ASOs for 1 week (1 µM) prior to exposure to vehicle (Veh) or Melph (2 µM) for 48 hours (left) or vehicle (Veh) or bortezomib (Bort; 5 nM) for 48 hours (right). Data are the mean ± S.D. from one representative experiment performed in triplicate. Statistically significant differences were detected using two-way ANOVA (*****P* <0.0001; ****P* <0.001; ***P* <0.01).

Supplementary Figure 1. ILF2-ASOs induce DNA damage activation and enhance MM cells' sensitivity to DNA-damaging agents (continue).

(**N**) Schematic of ASO and melphalan (Melph) treatment in MM xenografts. GFP^+Luc^+ KMS11 cells (2 x 10^6) were injected into sublethally irradiated NSG mice. Ten days after transplantation, mice were injected with luciferin and tumor burden was quantified using the IVIS Spectrum bioluminescence imaging system. Mice were randomized based on tumor burden (day 0). Mice were injected with NT or ILF2 ASOs (50 mg/kg) alone for 7 days prior to receiving NT or ILF2 ASOs (25 mg/kg) in combination with Melph (2.5 mg/kg) every other day for 3 doses by intraperitoneal injection. Tumor burden was evaluated using bioluminescence imaging at days 7 and 12. The BM and liver from each mouse were collected at day 12 and analyzed. (NT ASOs+Veh, n=17; NT ASOs+Melph, n=16; ILF2 ASOs+Veh, n=16; ILF2 ASOs+Melph, n=14; 2 independent experiments were performed and combined).
(**O**) Left, ILF2 expression in BM (left) and liver (right) biopsy specimens obtained from representative xenografts treated with NT ASOs+Veh, ILF2 ASOs+Veh, NT ASOs+Melph, and ILF2 ASOs+Melph. Scale bars represent 50 µm. Right, relative *ILF2* expression determined by real time PCR in GFP^+ KMS11 cells isolated from BM of mice treated with NT or ILF2 ASOs for the time of the experiment. The expression level of *ILF2* was normalized to that of *ACTIN*. (NT ASOs, n=9; ILF2 ASOs, n=11). Statistical significance was calculated using a 2-tailed Student t-test (****$P < 0.0001$).
(**P**) Cleaved caspase 3 expression in BM (left) and liver (right) biopsy specimens obtained from representative xenografts treated with NT ASOs+Veh, ILF2 ASOs+Veh, NT ASOs+Melph, and ILF2 ASOs+Melph. Scale bars represent 50 µm.
Source data are provided as a Source Data file.

Supplementary Figure 2

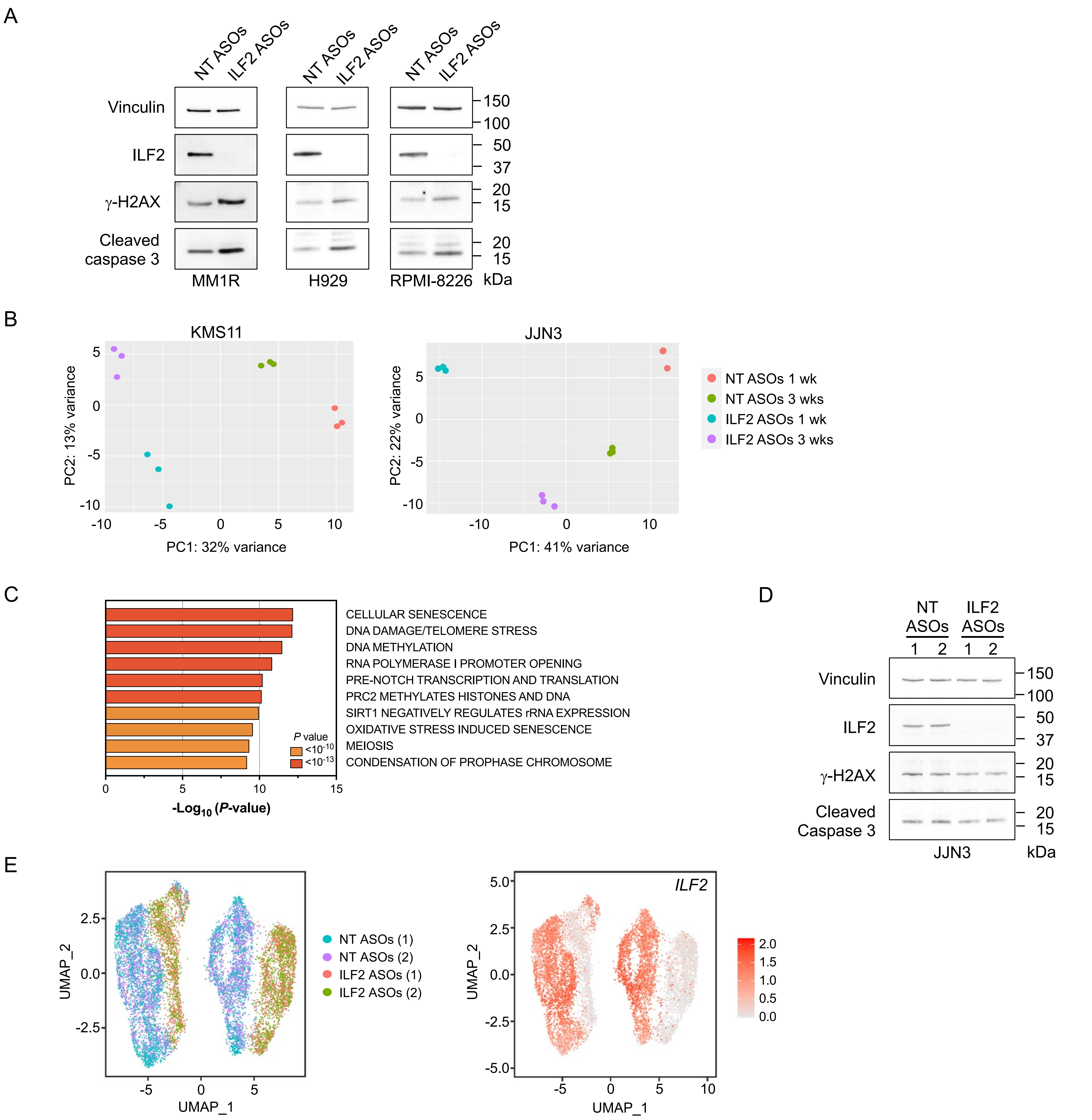

Supplementary Figure 2 (continue)

F

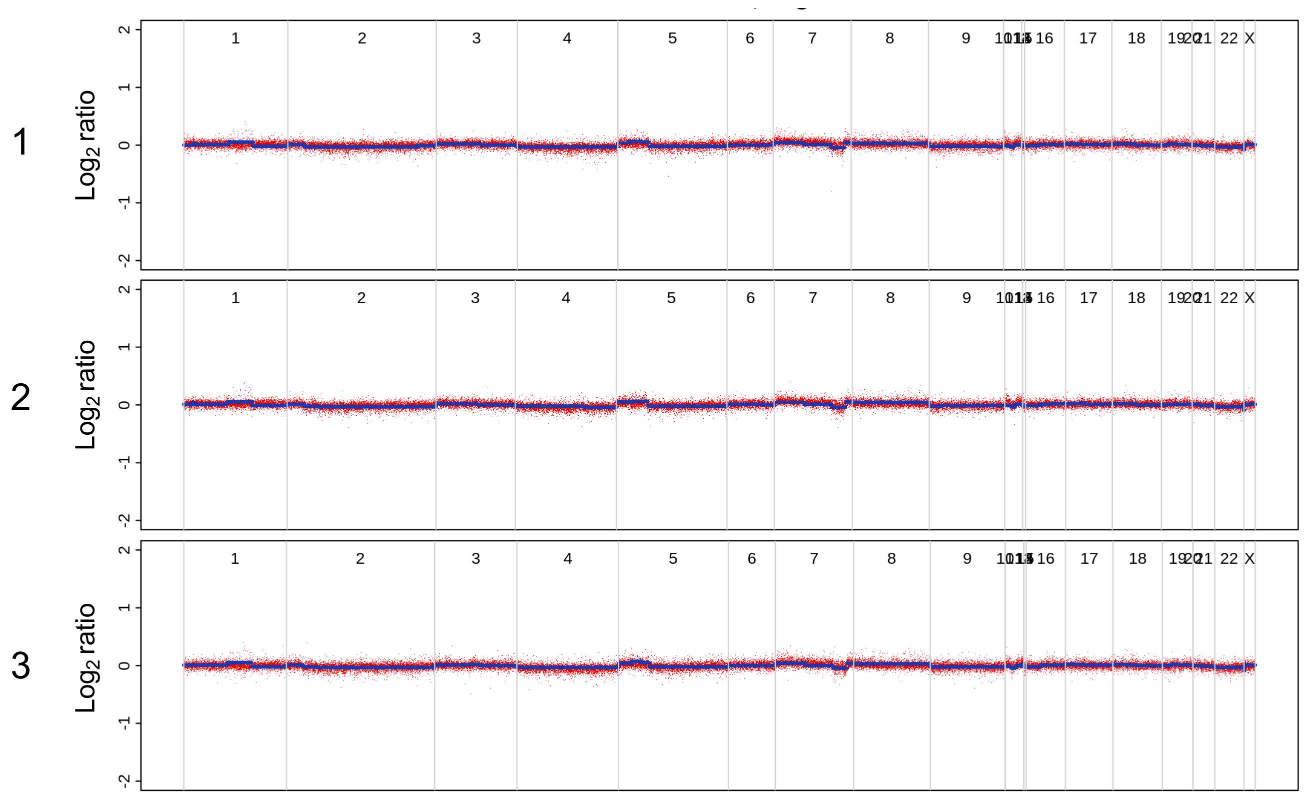

G

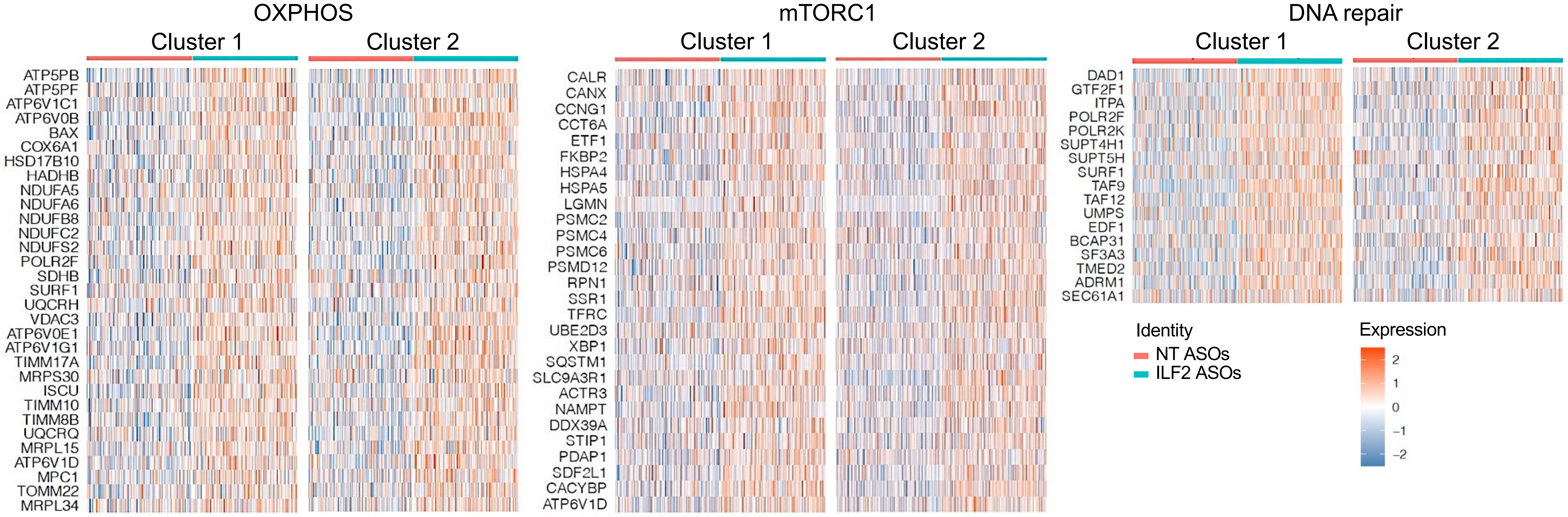

Supplementary Figure 2 (continue)

H

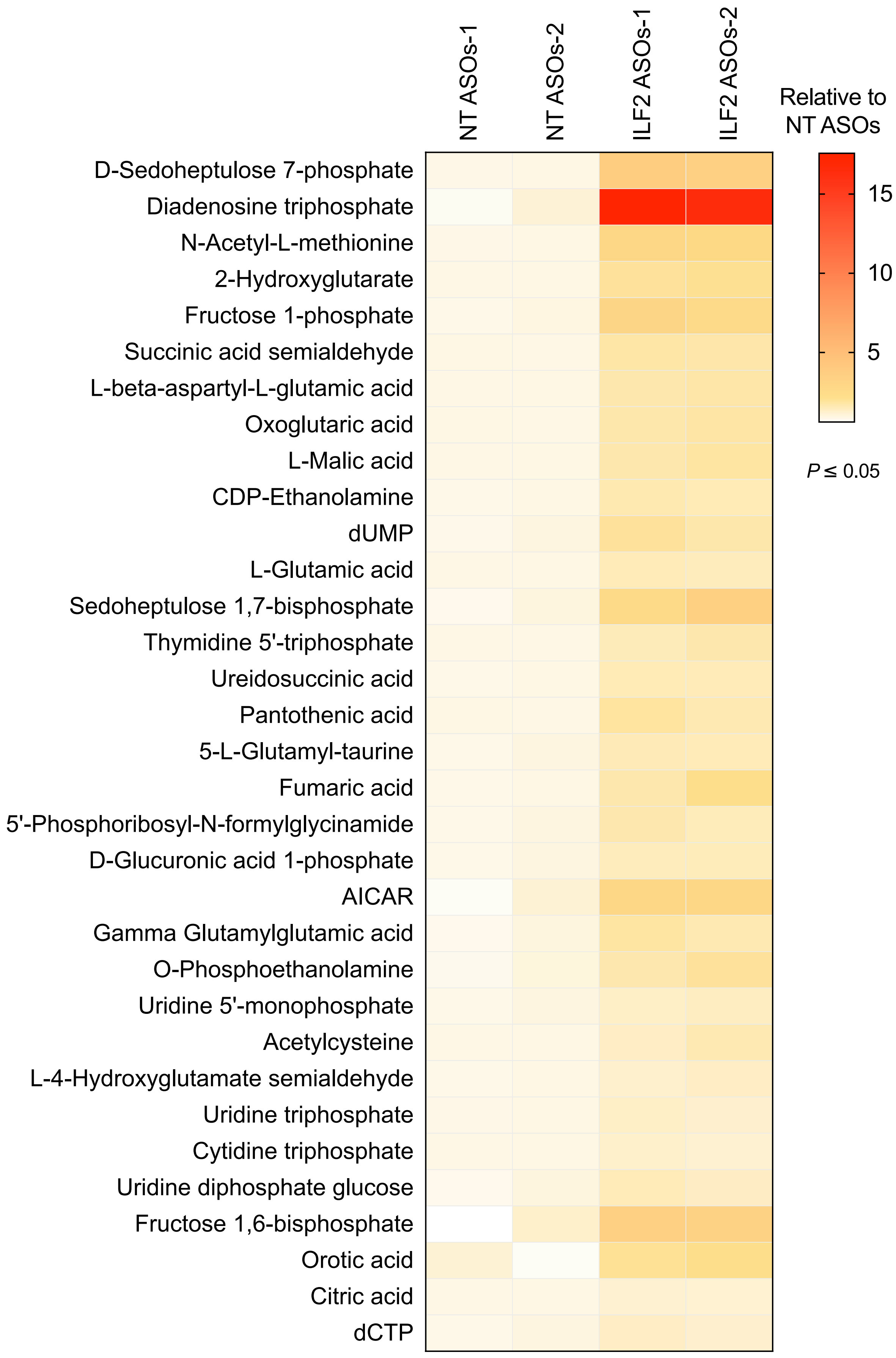

I

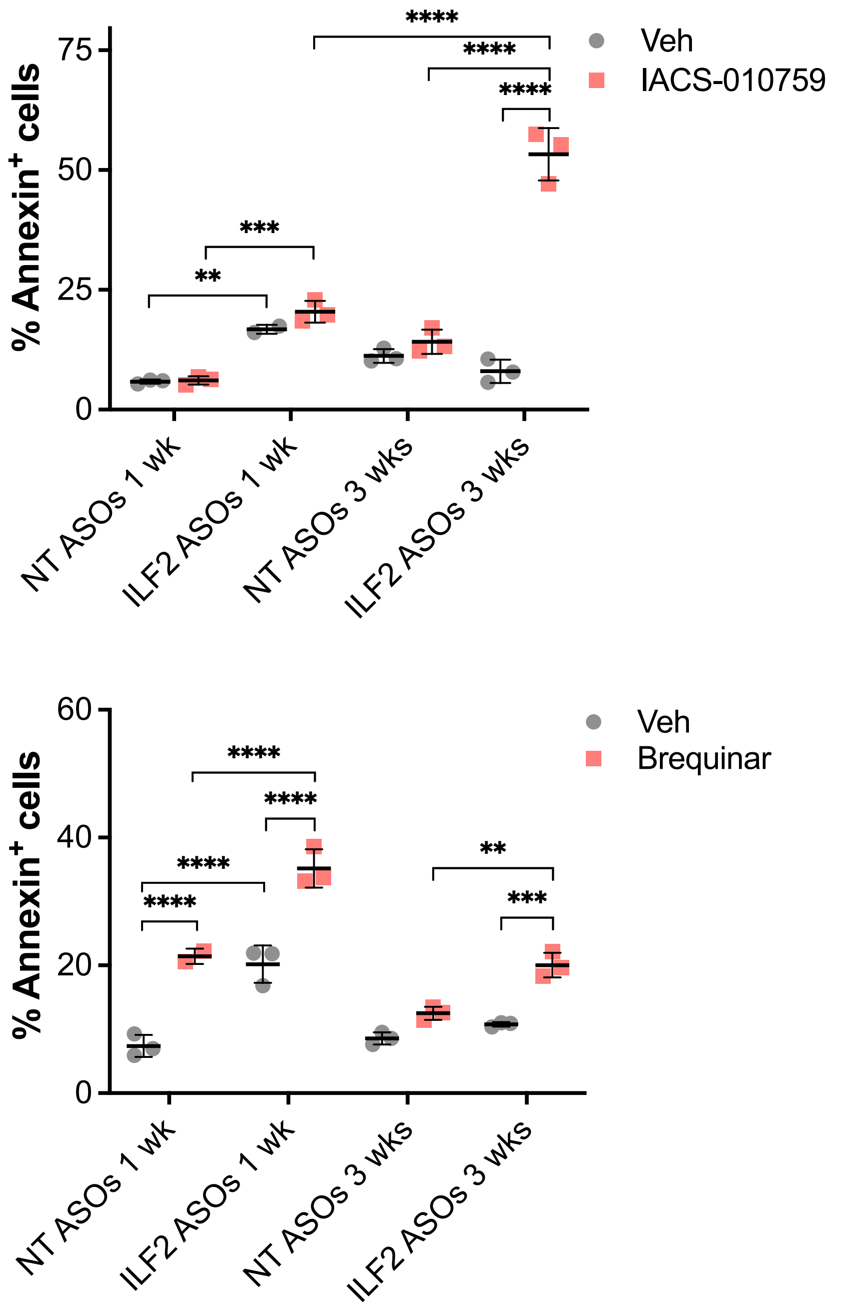

J

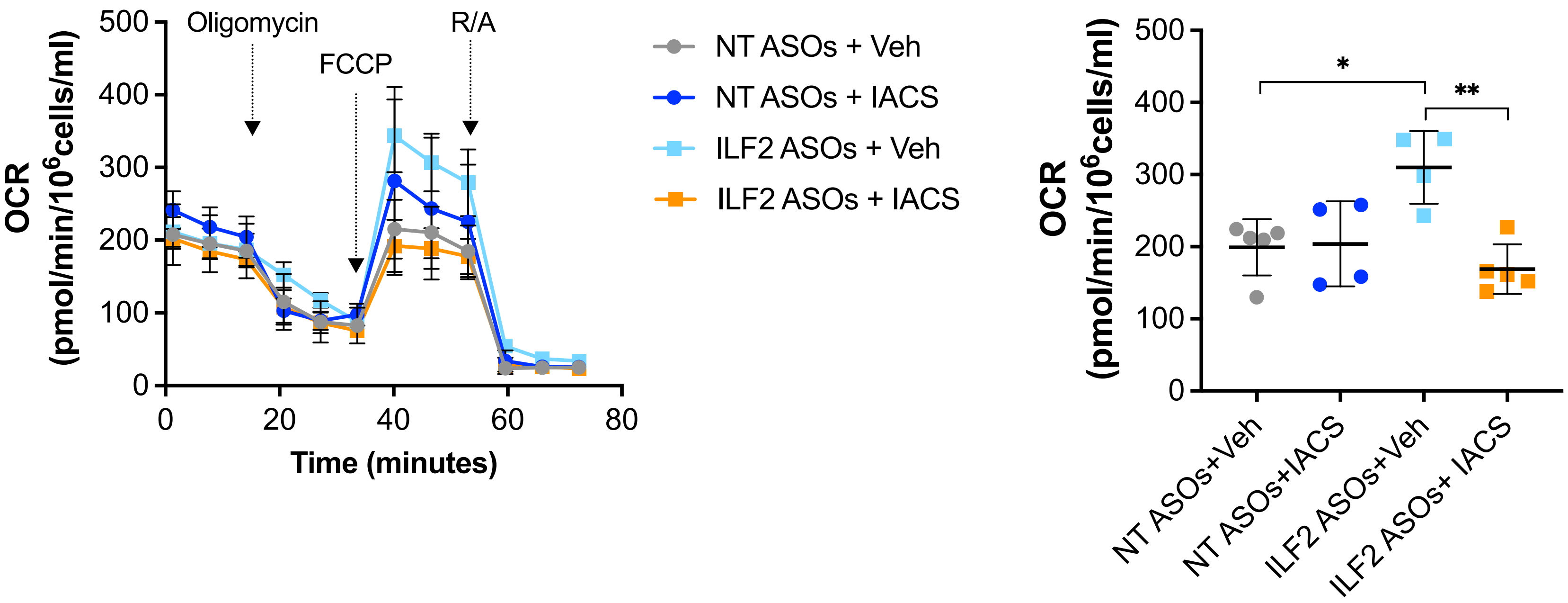

K

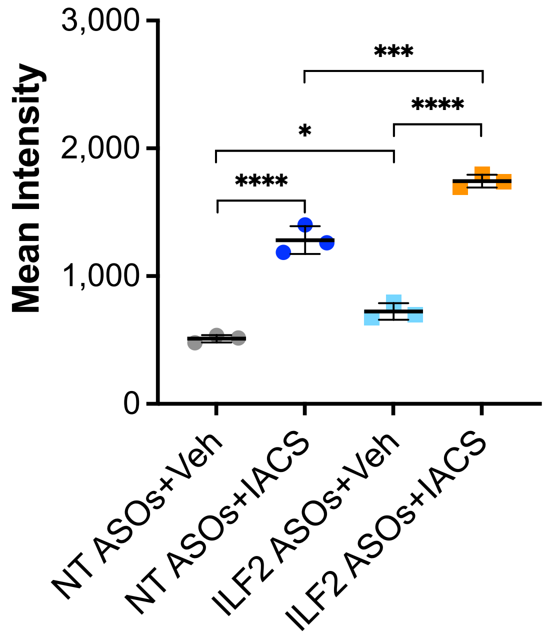

Supplementary Figure 2 (continue)

L

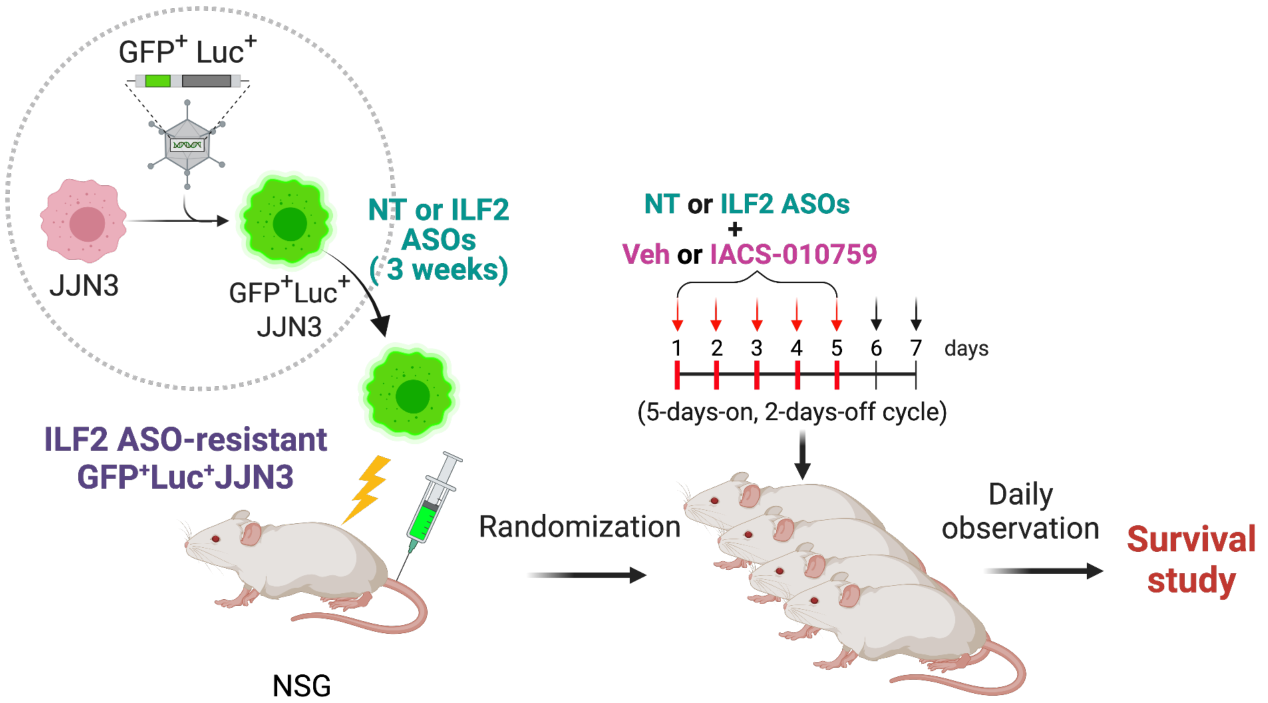

M

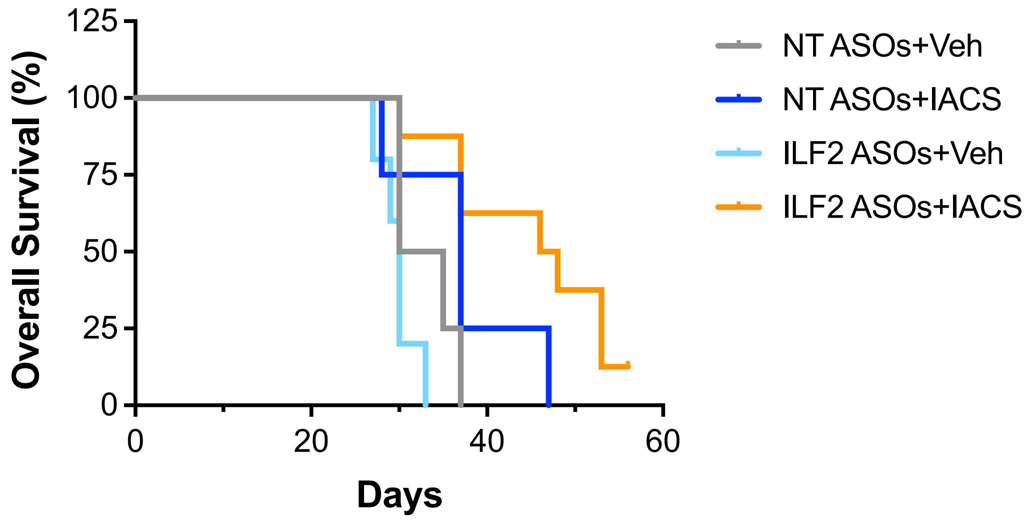

Supplementary Figure 2. Metabolic reprogramming mediates MM cells' resistance to DNA damage activation.

(A) Western blot analysis of ILF2, γH2AX and cleaved caspase 3 in MM1R (left), H929 (middle), and RPMI-8226 (right) cells after treatment with NT or ILF2 ASOs (1 μM, 2 μM and 1 μM, respectively) for 3 weeks. Vinculin was used as a loading control.
(**B**) Principal component analysis (PCA) of RNA-seq data from NT ASO– and ILF2 ASO–treated KMS11 (left) and JJN3 (right) cells at the indicated time points. Each treatment was performed in biological triplicates. Wk, week.
(**C**) Pathway enrichment analysis of genes that were significantly downregulated compared with the corresponding NT ASO-treated cells in JJN3 but not in KMS11 cells treated with ILF2 ASOs for 3 weeks as compared with those treated for 1 week (adjusted $P \leq 0.05$). The top 10 Gene Ontology gene sets are shown.
(**D**) Western blot analysis of ILF2, γH2AX, and cleaved caspase 3 in ILF2 ASO–resistant JJN3 cells after treatment with NT ASOs or ILF2 ASOs (1 μM) for 3 weeks (n=2 biological replicates; #1-2). Vinculin was used as a loading control.
(**E**) UMAP plots of scRNA-seq data from Fig. 2C showing single JJN3 cells after 3 weeks of NT or ILF2 ASOs (1 μM) treatment. Different colors represent the individual replicates (left) or *ILF2* expression levels (right). Red shading indicates normalized gene expression.
(F) Relative copy number alterations in JJN3 cells after 3 weeks of treatment with ILF2 ASOs (n=3 replicates) compared with those in JJN3 after treatment with NT ASOs (n=3 replicates). Log2 ratios of genome wide copy number are shown. Log2 = 0 means no significant difference between ILF2 ASO-treated cells and NT ASO-treated cells.
(**G**) Heatmaps of the genes belonging to the OXPHOS (left), mTORC1 (middle), or DNA repair pathways (right) that were significantly overexpressed in each of the 2 cell clusters shown in Fig. 2C after treatment with ILF2 ASOs for 3 weeks compared with those treated with NT ASOs.
(H) Heatmap of the 33 metabolites that were significantly enriched in JJN3 cells treated with ILF2 ASOs for 3 weeks compared with cells treated with NT ASOs (n=2 biological replicates; adjusted $P \leq 0.05$).
(**I**) Frequencies of apoptotic JJN3 cells after 1 week (wk) or 3 weeks (wks) of NT or ILF2 ASOs (1 μM) exposure followed by 72 hours of treatment with vehicle (Veh), IACS-010759 (1 μM; top) or brequinar (100 nM; bottom). Data are expressed as the mean ± S.D. from one representative experiment performed in triplicate. Statistically significant differences were detected using two-way ANOVA (****P <0.0001; ***P <0.001; **P <0.01).
(J) Oxygen consumption rates (OCRs; left panel) and maximal OCRs (right panel) in JJN3 cells treated with NT or ILF2 ASOs (1 μM) for 3 weeks prior to receiving ASOs alone or in combination with vehicle (Veh) or IACS-010759 (IACS; 1 μM for 72 hours). Each data point is the mean ± S.D. of 4 replicates. FCCP, carbonyl cyanide-p-trifluoromethoxy-phenylhydrazone; R/A, rotenone/antimycin. Statistically significant differences were detected using two-way ANOVA (**P <0.01; *P <0.05).
(**K**) ROS production in JJN3 cells treated with NT or ILF2 ASOs (1 μM) for 3 weeks prior to receiving 1 μM IACS-010759 (IACS) for 48 hours. Data are expressed as the mean ± S.D. from one representative experiment performed in triplicate. Statistically significant differences were detected using two-way ANOVA (****P <0.0001; ***P <0.001; *P <0.05).
(L) Schematic of ASO and IACS-010759 treatments in MM xenografts. ILF2 ASO–resistant GFP^+Luc^+ JJN3 cells (1×10^6) were injected into NSG mice. Five days after transplantation, mice were randomized into 4 groups and treated with NT or ILF2 ASOs alone (25 mg/kg) or in combination with IACS-010759 (IACS; 10 mg/kg) on a 5-days-on, 2-days-off cycle until they were euthanized because they were moribund.
(M) Survival curves of NSG mice that received transplants of ILF2 ASO-resistant JJN3 cells after receiving NT or ILF2 ASOs alone (NT ASOs+Veh or ILF2 ASOs+Veh) or in combination with IACS-010759 (IACS; 10 mg/kg) (NT ASOs+Veh, n=4; NT ASOs+IACS, n=4; ILF2 ASOs+Veh, n=6; ILF2 ASOs+IACS, n=8). Survival curves were analyzed using the Mantel–Cox log-rank test (ILF2 ASOs+Veh vs ILF2 ASOs+IACS: $P = 0.0012$; ILF2 ASOs+IACS vs NT ASOs+IACS: $P = 0.10$; NT ASOs+Veh vs NT ASOs+IACS: $P = 0.24$).
Source data are provided as a Source Data file.

Supplementary Figure 3

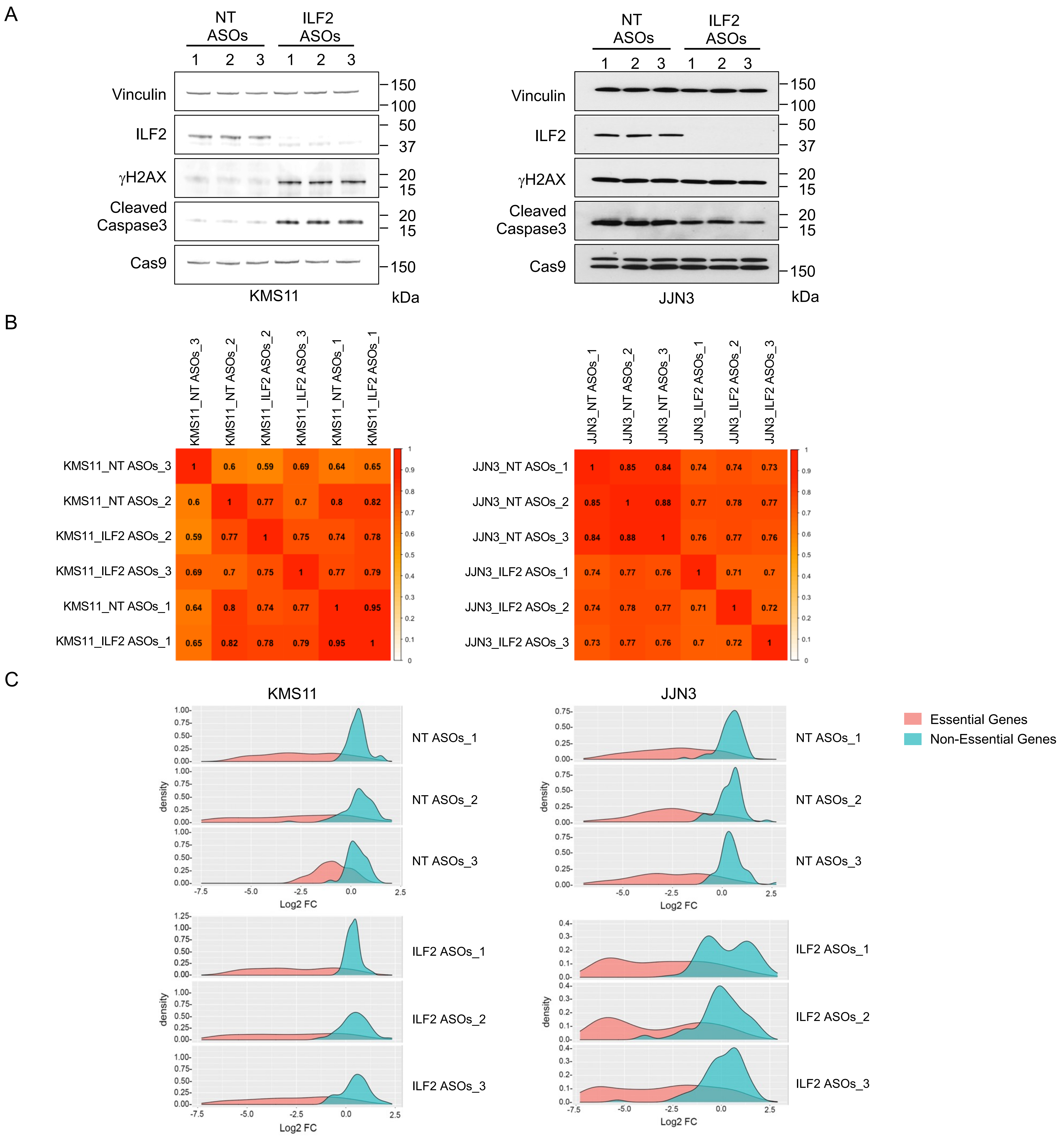

Supplementary Figure 3 (continue)

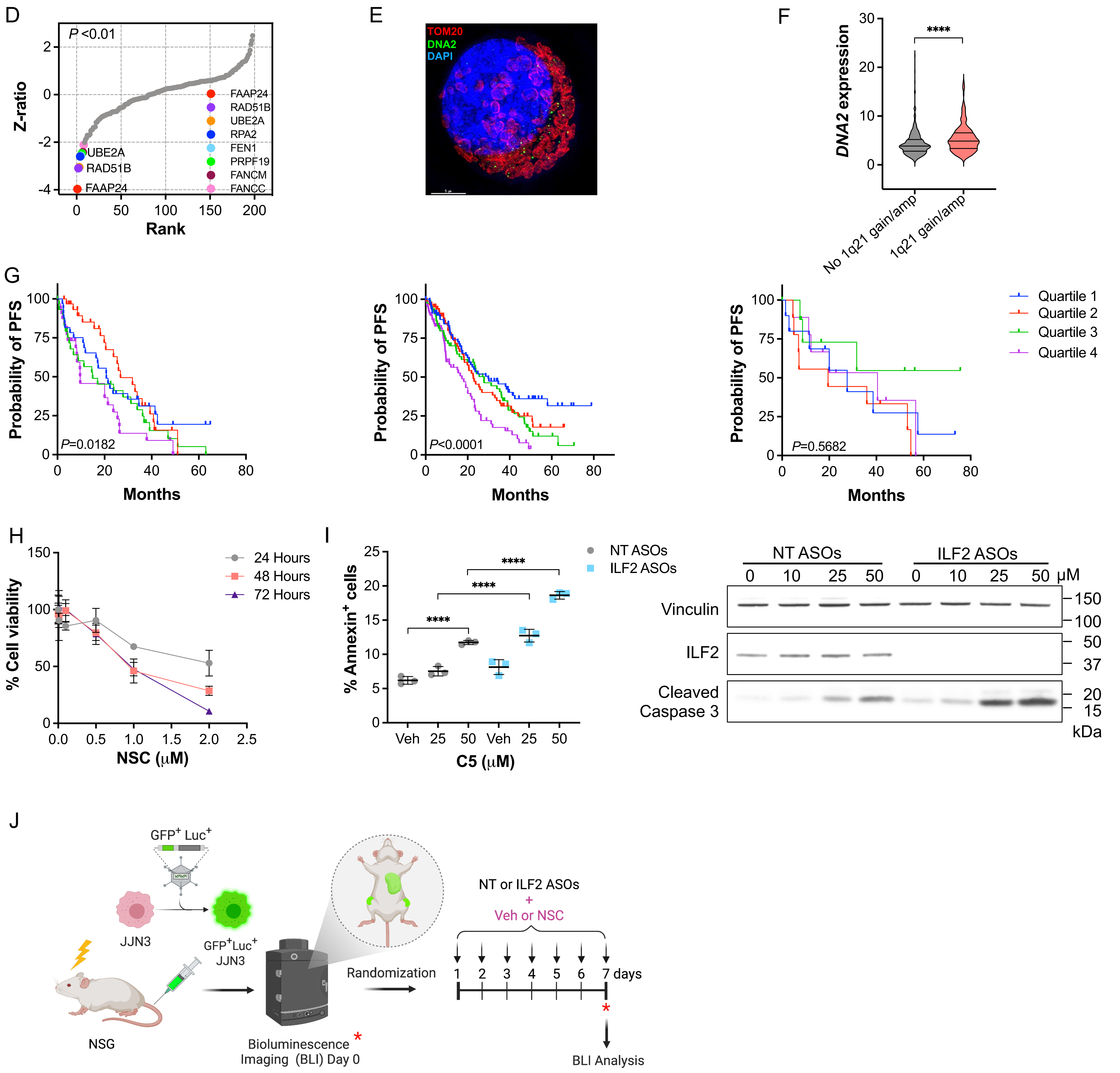

Supplementary Figure 3. DNA2 is essential for maintaining MM cells' survival after DNA damage–induced metabolic reprogramming.

(**A**) Western blot analysis of ILF2, γH2AX, cleaved caspase 3 and Cas9 in NT ASO– or ILF2 ASO–treated KMS11 (left) and JJN3 (right) cells (0.5 μM and 1 μM, respectively) after 3 weeks of culture. The 3 biological replicates from the experiment described in Fig. 3A are shown (#1-3). Vinculin was used as a loading control.
(**B**) Correlation of the sgRNAs' gene-level $\log_2$ fold changes in KMS11 (left) and JJN3 (right) cells among the 3 independent sets of experiments.
(**C**) Density functions of gene-level $\log_2$ fold changes (FC) of essential and non-essential genes in KMS11 (left) or JJN3 (right). Samples were collected after 3 weeks of NT (top) or ILF2 (bottom) ASOs treatment.
(**D**) Ranking of DNA repair genes whose sgRNAs were significantly depleted in ILF2 ASO–treated KMS11 cells as compared with NT ASO–treated cells. The inset shows genes on the top ranks (adjusted P <0.01).
(**E**) Representative immunofluorescence image of DNA2 in JJN3 cells. Image was captured and processed using a Delta Vision OMX V4 Blaze Super-Resolution System. Green indicates DNA2; red, TOM20 (mitochondrial marker); and blue, DAPI. Scale bar represents 5 μm.
(**F**) Violin plot of RNA-seq based *DNA2* expression in the PCs of newly diagnosed MM patients (n=543). Samples were divided into 2 groups (with or without the 1q21 gain or amplification). The lines inside each violin plot define the 4 quartiles of *DNA2* expression. Statistically significant differences were detected using a 2-tailed Student t-test (****P <0.0001).
(**G**) Kaplan–Meier plots of progression-free survival (PFS) according to *DNA2* expression in MM PCs from the IA16 CoMMpass dataset from the Multiple Myeloma Research Foundation. Quantile 1 includes samples with the lowest expression of *DNA2*. Quantile 4 includes samples with the highest expression of *DNA2*. Shown are the median PFS durations of patients who received PIs alone (n=129; left; P=0.0182); patients who received PIs in combination with other therapies (n=326; middle; P<0.0001); and patients who received immunomodulatory drugs (n=37; right; P=0.5682).
(**H**) Frequencies of live JJN3 cells after treatment with NSC at the indicated concentrations for 24, 48, and 72 hours. Data from one representative experiment performed in triplicates are expressed as the mean frequencies ± S.D. of live cells among all cells at each timepoint. Live cells were counted using Trypan Blue staining.
(**I**) Left, frequencies of apoptotic (annexin V^+) JJN3 cells after 3 weeks of NT ASO or ILF2 ASO (1 μM) exposure followed by 48 hours of treatment with vehicle (Veh) or the DNA2 inhibitor C5 (C5) at the indicated concentrations. Data are expressed as the mean ± S.D. from one representative experiment performed in triplicates. Statistically significant differences were detected using two-way ANOVA (****P <0.0001). Right, Western blot analysis of ILF2 and cleaved caspase 3 in JJN3 cells treated with NT or ILF2 ASOs alone or in combination with C5 at the indicated concentrations for 48 hours. Vinculin was used as a loading control.
(**J**) Schematic of ASOs and NSC treatments in MM xenografts. ILF2 ASO–resistant GFP^+Luc^+ JJN3 cells (1×10^6) were injected into NSG mice. Ten days after transplantation, mice were injected with luciferin, and tumor burden was quantified using the IVIS Spectrum bioluminescence imaging system. Mice were randomized into 4 groups based on tumor burden on day 0. Mice were injected with NT or ILF2 ASOs alone (25 mg/kg) or in combination with NSC (10 mg/kg) every day for 7 days. Tumor burden was evaluated by bioluminescence imaging on days 0 and 7. Source data are provided as a Source Data file.

Supplementary Figure 4

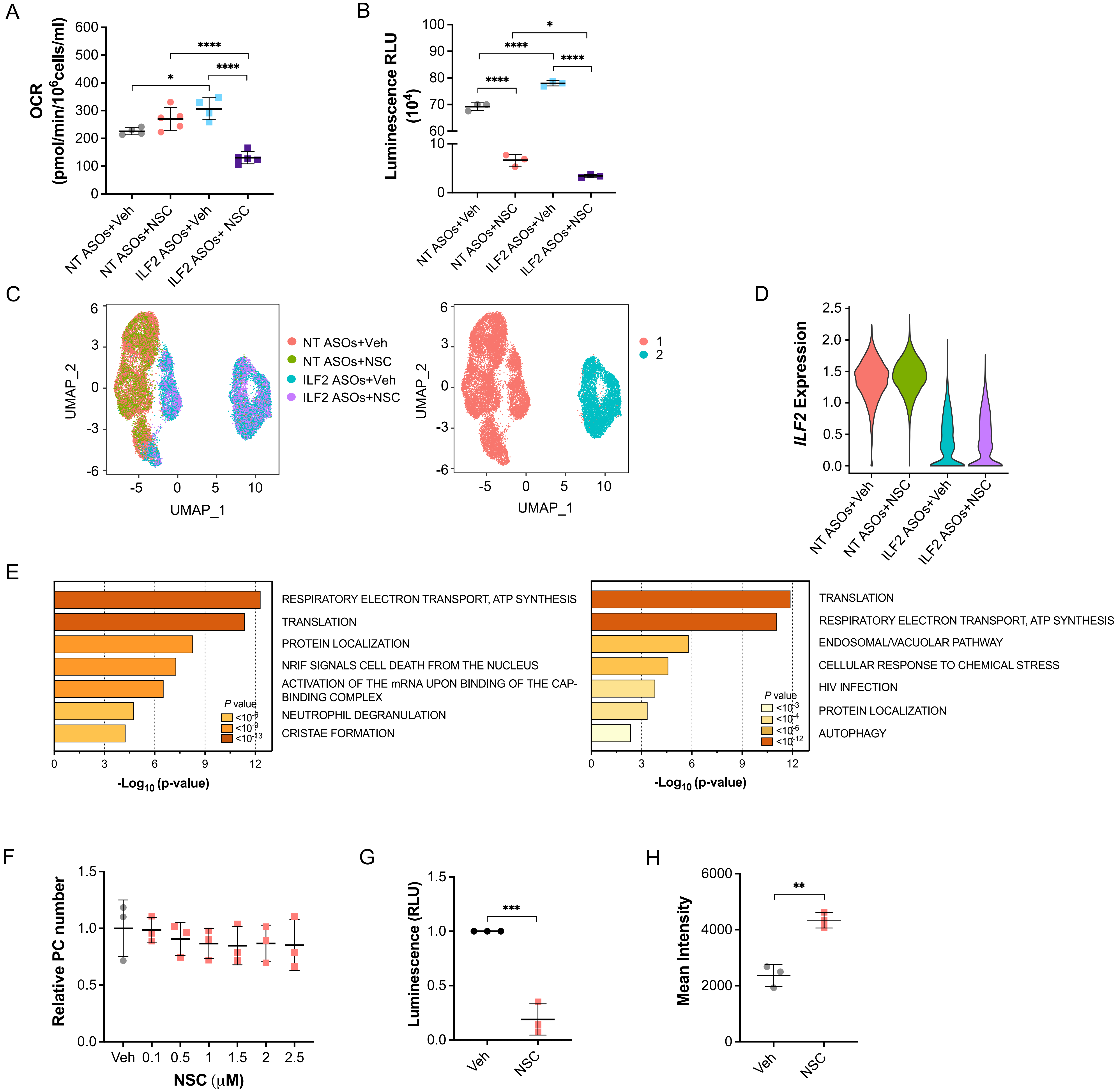

Supplementary Figure 4 (continue)

I

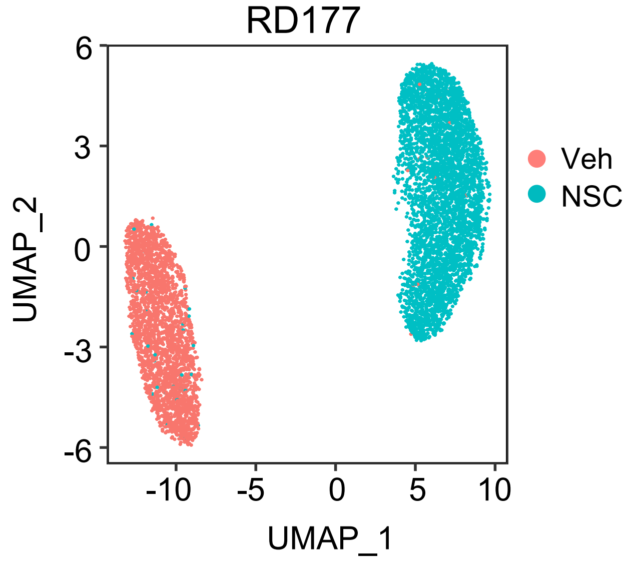

J

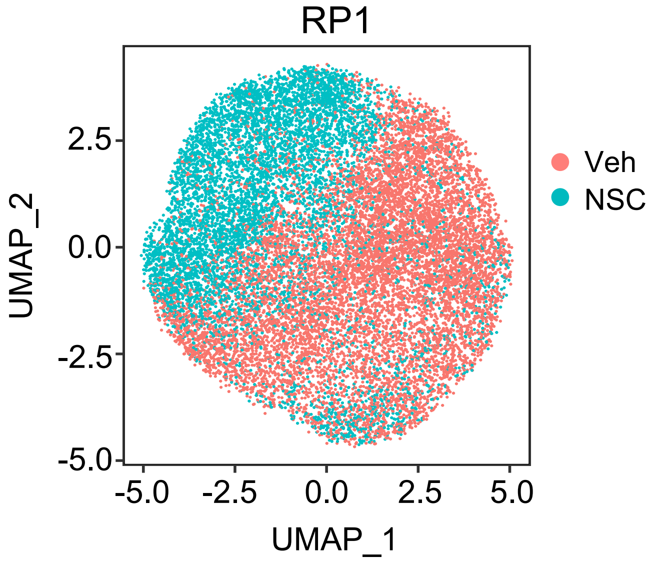

K

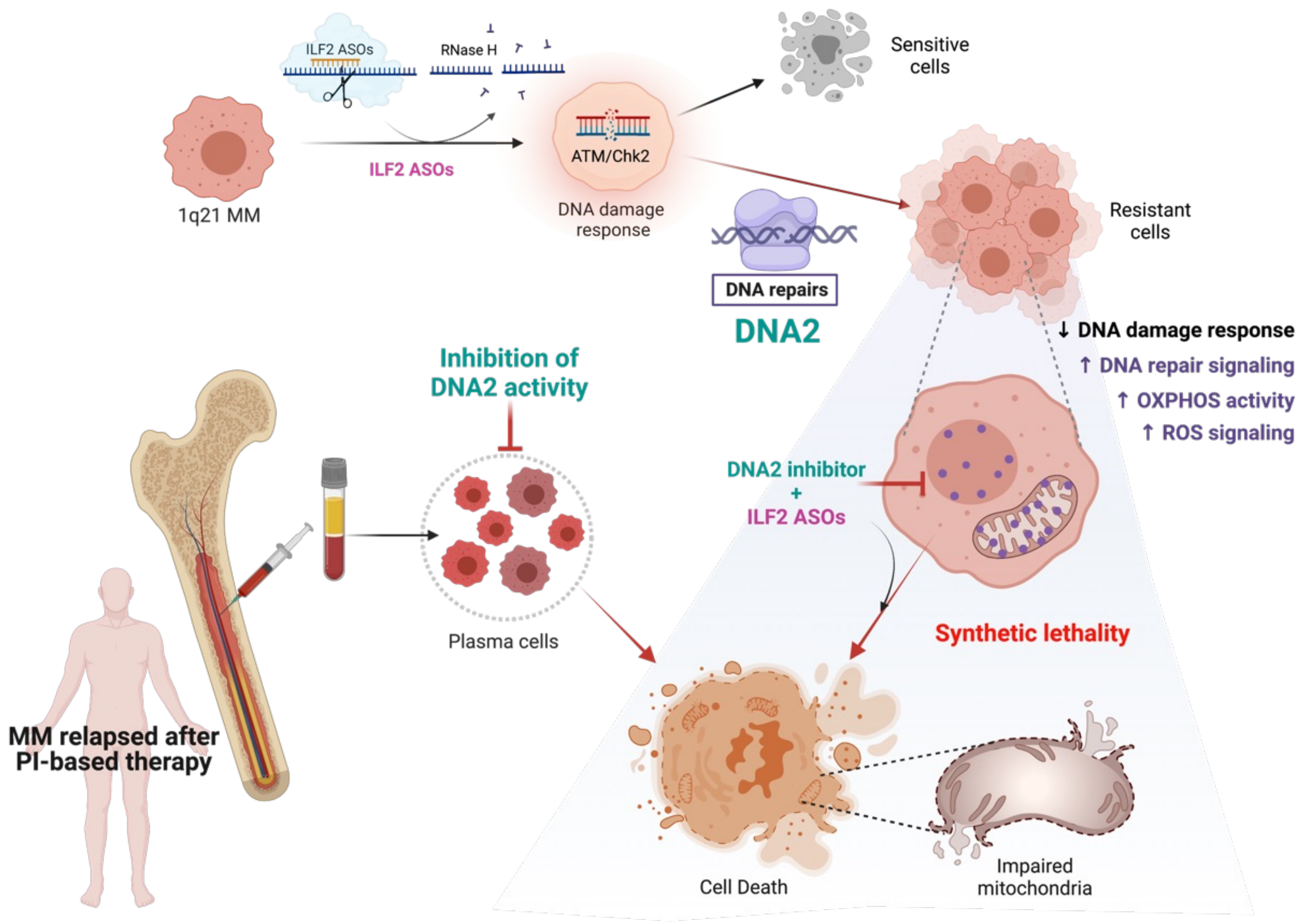

Supplementary Figure 4. DNA2 is essential for lowering mitochondrial ROS production in MM cells.

(**A**) Maximal OCRs in JJN3 cells treated with NT or ILF2 ASOs (1 μM) for 3 weeks prior to receiving ASOs alone or in combination with 1 μM NSC for 72 hours. The mean ± S.D. from at least 4 replicates per group are shown. Statistically significant differences were detected using two-way ANOVA (****P <0.0001; *P <0.05).
(**B**) NAD/NADH quantifications in JJN3 cells treated with NT or ILF2 ASOs (1 μM) for 3 weeks prior to receiving 1 μM NSC for 48 hours. Data are expressed as the mean ± S.D. of Relative Light Unit (RLU) from one representative experiment performed in triplicate. Statistically significant differences were detected using two-way ANOVA (****P <0.0001; *P <0.05).
(**C**) UMAPs plot of scRNA-seq data displaying pooled single JJN3 cells from 2 independent experiments after 3 weeks of NT or ILF2 ASOs (1 μM) treatment prior to receiving ASOs alone (n=5,940 cells and n=4,790 cells, respectively) or in combination with 1 μM NSC for 48 hours (n=4,971 cells and n=5,317 cells, respectively). Different colors represent the sample origins (left) and the 2 identities of the main cluster (right).
(**D**) Violin plots showing the distribution of *ILF2* expression values across the 4 samples shown in Fig. S4C.
(**E**) Pathway enrichment analyses of significantly upregulated genes in JJN3 cells treated with ILF2 ASOs plus NSC as compared with cells treated with ILF2 ASOs alone in the major clusters 1 (left) or 2 (right) shown in Fig. S4C (adjusted $P \leq 0.05$). Reactome gene sets are shown. No differences between the expression profile of JJN3 cells treated with NT ASOs + NSC and that of the cells treated with NT ASOs alone (NT ASOs + Veh) were detected.
(**F**) Number of PCs isolated from 2 healthy donors' BM samples after treatment with NSC. PCs were combined and treated with NSC at the indicated concentrations for 48 hours over a layer of mesenchymal cells. The experiment was performed in triplicate. Data were normalized to the vehicle (Veh)-treated control. The mean ± S.D. are shown. No statistical significance was detected using one-way ANOVA.
(G) NAD/NADH quantifications in PCs from the BM of MM patients with PI-based therapy failure (n=3) after treatment with vehicle (Veh) or 2 μM NSC for 48 hours over a layer of mesenchymal cells. Data were normalized to each sample's Veh-treated control and expressed as the mean ± S.D. of Relative Light Units (RLUs). Statistically significant differences were detected using a 2-tailed Student *t*-test (***P <0.001).
(H) ROS production in PCs from the BM of one MM patient with PI-based therapy failure after treatment with vehicle (Veh) or NSC (2 μM) for 48 hours over a layer of mesenchymal cells. Data are expressed as the mean ± S.D. of one representative experiment performed in triplicate. Statistically significant differences were detected using a 2-tailed Student *t*-test (**P <0.01).
(**I**) UMAP of scRNA-seq data displaying PCs from one MM patient with 1q21 amplification (RD177), whose disease failed PI-based therapy. PCs were treated for 48 hours with vehicle (Veh) or 2 μM NSC over a layer of mesenchymal cells. Different colors represent the sample origins.
(**J**) UMAP of scRNA-seq data displaying PCs from one MM patient (RP1) without 1q21 amplification, whose disease failed PI-based therapy. PCs were treated for 48 hours with vehicle (Veh) or 2 μM NSC over a layer of mesenchymal cells. Different colors represent the sample origins.
(**K**) Proposed working model. Resistance to DNA damage induced by ILF2 depletion in 1q21 MM cells relies on metabolic reprogramming which switches MM cells' metabolism from glycolysis to high mitochondrial energy demand. Targeting DNA2 activity induces synthetic lethality in metabolically reprogrammed MM cells, such as those that have acquired resistance to PI-based therapy.
Source data are provided as a Source Data file.

Supplementary Table 1: Sequences of the ASOs targeting ILF2 used in this study

ID	Sequence	Species
1072134	GTTTACAGCATTTCAA	Mouse
1072178	AGCATTAACAGTTACG	Mouse
1072209	CTTAGATTATTAGTGC	Mouse
1146777	CCATGTAAAGCCCAGT	Human
1146797	GATGGATGGCATAGGT	Human
1146799	TATAGATGGATGGCAT	Human
1146809	AGTATTAGTTGAGAGA	Human
1146953	GTCATTAAGGTCTAAG	Human

Supplementary Table 2: Patient characteristics.

Sample ID	Gender	Age	Race/Ethicity	Sample Status	Previous Treatment	Genetic alterations	Amp 1q21	Analysis performed
RP1	Male	64	Hispanic	Relapsed MM	Ninlaro	t(4;14), t(11;14)	No	NSC treatment/scRNA-seq/NAD
RP4	Male	82	White	Relapsed MM	Bortezomib	monosomy 13	No	NSC treatment
RD177	Male	78	White/European	Relapsed MM	Bortezomib	normal karyotype	Yes	NSC treatment/scRNA-seq/NAD/ROS
RD192	Male	73	White/European	Relapsed MM	Bortezomib	normal karyotype	Yes	NSC treatment/scRNA-seq
RD190	Female	75	White/European	Relapsed MM	Bortezomib	normal karyotype	No	NSC treatment
RD103	Female	82	White/European	Relapsed MM	Bortezomib, Melphalan, and	Hyperdiploid karyotype	Yes	NSC treatment/NAD
M4-3251	Female	63	White	Relapsed MM	Bortezomib	t(4;14)	Yes	NSC treatment

Amp = anplification; NSC=NSC105808

Supplementary Table 3: Cell surface marker expression panel used for the identification, quantification, and purification of MM or plasma cells by flow cytometry.

**Xenografts**				
**Population**	**Gating strategy**			
Live cells	Single cells/DAPI$^-$			
Mouse cells	mCD45$^+$			
MM cells	GFP$^+$			
**Sorting MM cells**	**Single cells/DAPI$^-$/mCD45$^-$/GFP$^+$**			
**Flow cytometer setting**				
**BD Influx Cell Sorter (Xenografts)**				
**Laser color**	**Laser, nm**	**Band, nm/range**	**Fluorochrome**	**Marker**
UV	355	460/50 BP	DAPI	Viability
Red	633	750 BP	APC-Cy7	mCD45
Blue-green	488	530/40 BP	GFP	MM

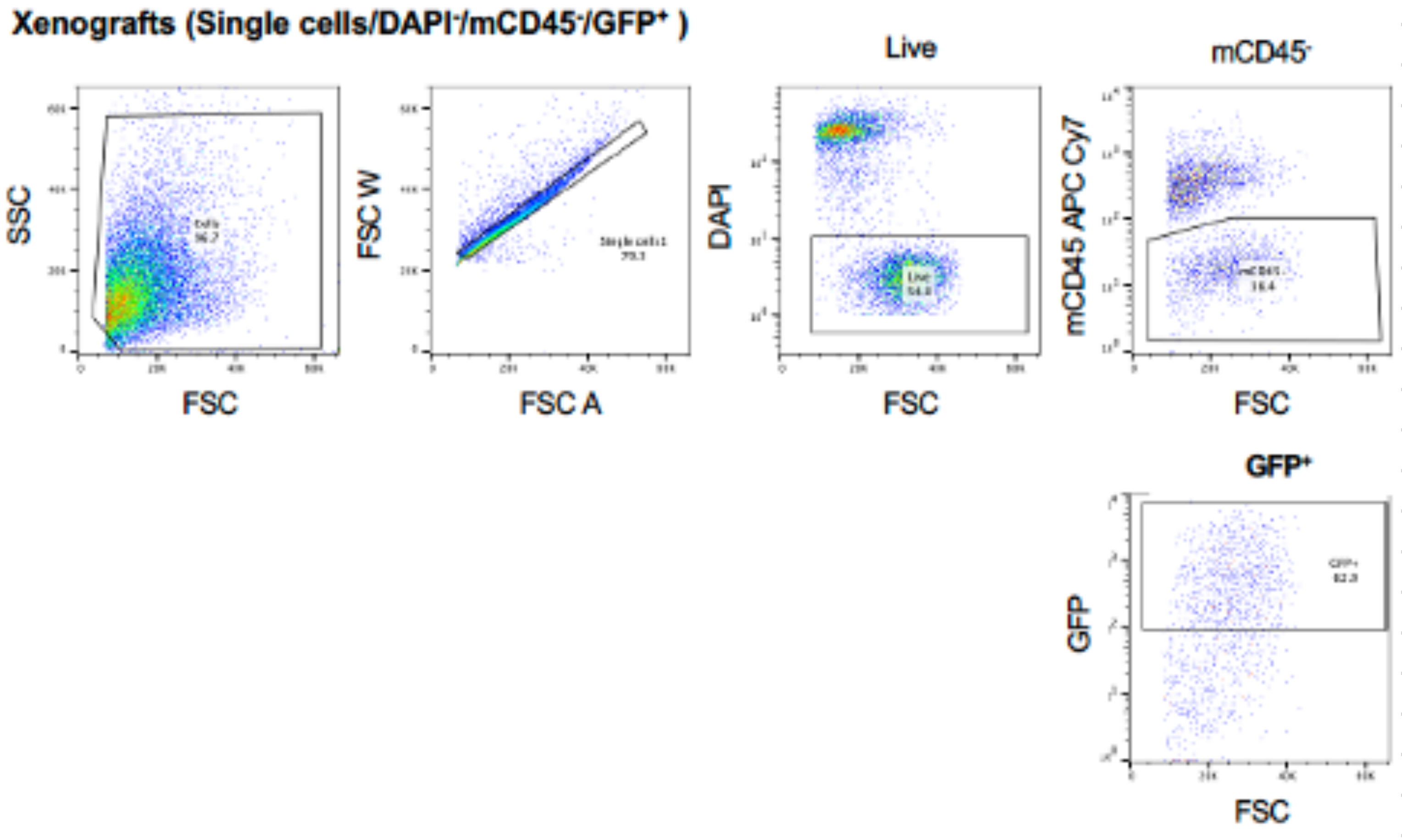

Supplementary Table 3: Cell surface marker expression panel used for the identification, quantification, and purification of MM or plasma cells by flow cytometry (continue).

**Primary cells**				
**Population**	**Gating strategy**			
Live cells		Single cells/DAPI$^-$		
hMSC	CD90$^+$			
Plasma cells	CD138$^+$			
**Sorting plasma cells**		**Single cells/DAPI$^-$/CD90$^-$/CD138$^+$**		
**Flow cytometer setting**				
		**BD Influx Cell Sorter (Primary samples)**		
**Laser color**	**Laser, nm**	**Band, nm/range**	**Fluorochrome**	**Marker**
UV	355	460/50 BP	DAPI	Viability
Blue-green	488	710/50 BP	PerCP-Cy5.5	CD90
Yellow	633	670/30 BP	APC	CD138

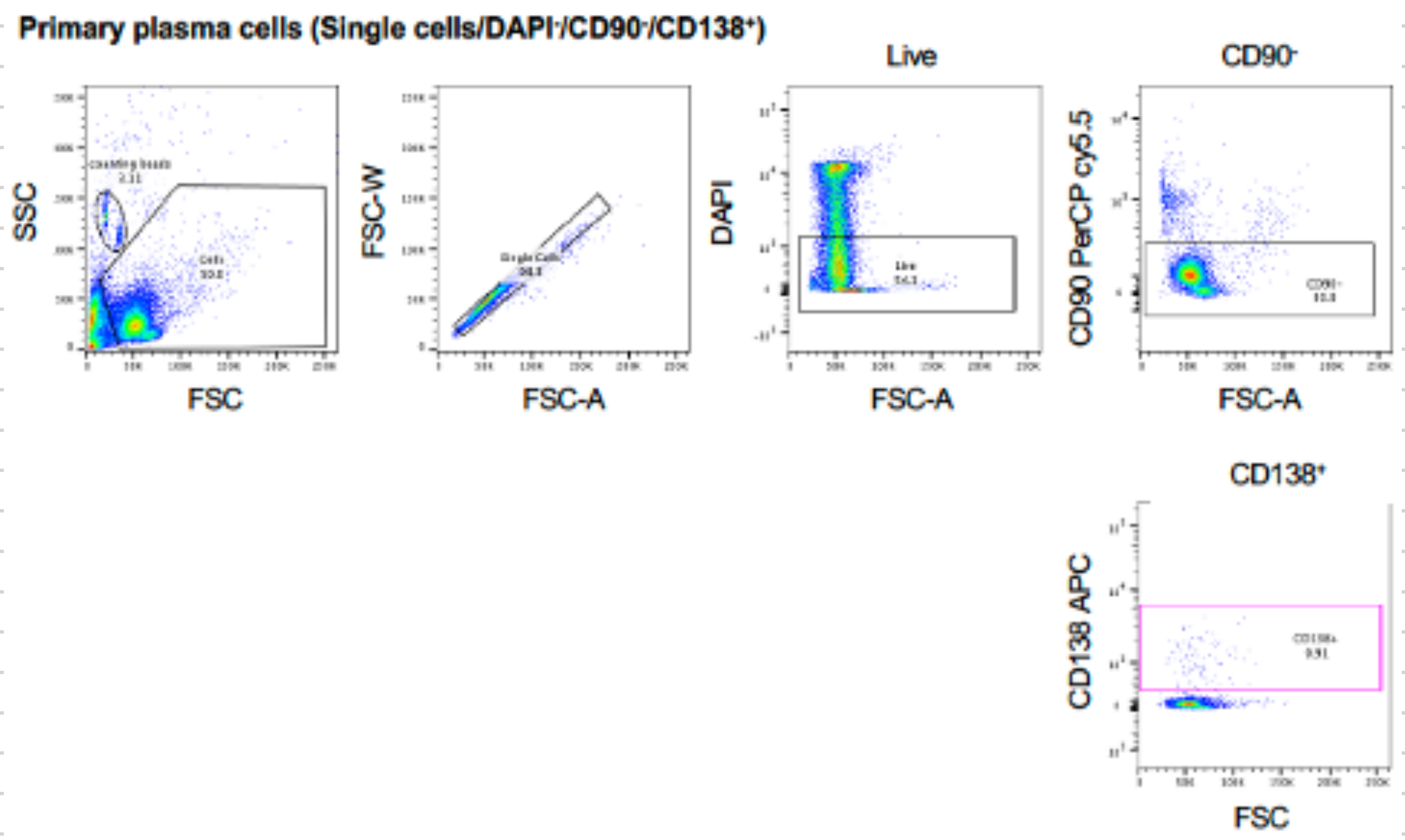

**MM cell lines**				
**Population**	**Gating strategy**			
Live cells	Single cells/DAPI$^-$			
MM cells	CD138$^+$			
**Sorting MM cells**	**Single cells/DAPI$^-$/CD138$^+$**			
**Flow cytometer setting**				
		**BD Influx Cell Sorter (MM cell lines)**		
**Laser color**	**Laser, nm**	**Band, nm/range**	**Fluorochrome**	**Marker**
UV	355	460/50 BP	DAPI	Viability
Yellow	633	670/30 BP	APC	CD138

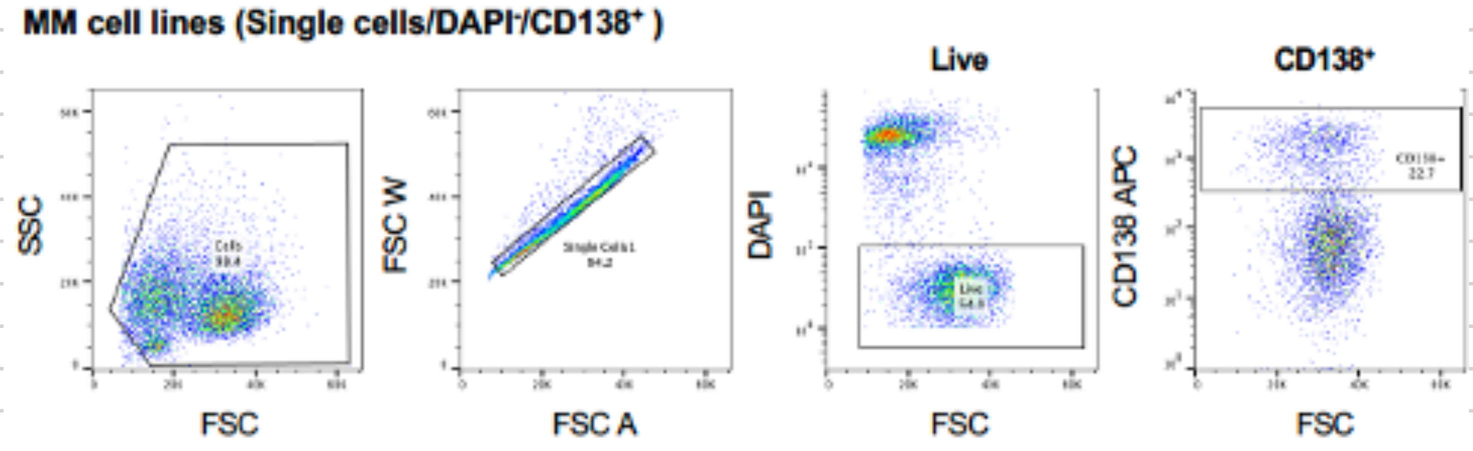

Supplement: Supplementary file 1 — Supplementary Information [file 41467_2024_45350_MOESM1_ESM.pdf]
