# Supplementary material for: Targeting DNA2 overcomes metabolic reprogramming in multiple myeloma
Source: Nat Commun. 2024 Feb 8;15:1203. doi: 10.1038/s41467-024-45350-8 (PMC10853245; doi:10.1038/s41467-024-45350-8)
Supplement: Supplementary file 3 — Description of additional Files [file 41467_2024_45350_MOESM3_ESM.docx]

**Description of Additional Supplementary Files**

Supplementary Data 1

Description: List of sgRNAs included in the CRISPR/Cas9 library screening
