# Supplementary material for: Targeting DNA2 overcomes metabolic reprogramming in multiple myeloma
Source: Nat Commun. 2024 Feb 8;15:1203. doi: 10.1038/s41467-024-45350-8 (PMC10853245; doi:10.1038/s41467-024-45350-8)
Supplement: Supplementary file 5 — Reporting Summary [file 41467_2024_45350_MOESM5_ESM.pdf]

nature portfolio

Corresponding author(s): Simona Colla
Last updated by author(s): Nov 30, 2023

Reporting Summary

Nature Portfolio wishes to improve the reproducibility of the work that we publish. This form provides structure for consistency and transparency in reporting. For further information on Nature Portfolio policies, see our Editorial Policies and the Editorial Policy Checklist.

Statistics

For all statistical analyses, confirm that the following items are present in the figure legend, table legend, main text, or Methods section.

n/a	Confirmed	
☐	☒	The exact sample size (n) for each experimental group/condition, given as a discrete number and unit of measurement
☐	☒	A statement on whether measurements were taken from distinct samples or whether the same sample was measured repeatedly
☐	☒	The statistical test(s) used AND whether they are one- or two-sided<br>*Only common tests should be described solely by name; describe more complex techniques in the Methods section.*
☒	☐	A description of all covariates tested
☒	☐	A description of any assumptions or corrections, such as tests of normality and adjustment for multiple comparisons
☐	☒	A full description of the statistical parameters including central tendency (e.g. means) or other basic estimates (e.g. regression coefficient) AND variation (e.g. standard deviation) or associated estimates of uncertainty (e.g. confidence intervals)
☐	☒	For null hypothesis testing, the test statistic (e.g. F, t, r) with confidence intervals, effect sizes, degrees of freedom and P value noted<br>*Give P values as exact values whenever suitable.*
☒	☐	For Bayesian analysis, information on the choice of priors and Markov chain Monte Carlo settings
☒	☐	For hierarchical and complex designs, identification of the appropriate level for tests and full reporting of outcomes
☒	☐	Estimates of effect sizes (e.g. Cohen's d, Pearson's r), indicating how they were calculated

Our web collection on statistics for biologists contains articles on many of the points above.

Software and code

Policy information about availability of computer code

Data collection	Flow cytometry data were acquired using the BD FACSDiva (version 8.01). Immunofluorescence images were obtained using a confocal microscope (Nikon Instruments Inc) and the Delta Vision OMX Blaze V4 Super Resolution system.
Data analysis	GraphPad Prism 10.1.0 was used to create the graphs (https://www.graphpad.com). BD FACSDiva version 8.01 (https://www.bdbiosciences.com/en-ca/products/software/instrument-software/bd-facsdiva-software) and FlowJo version 10.10.0 (www.flowJo.com) were use for FACS and flow cytometry analyses, respectively. Metascape (https://metascape.org) was used for pathway enrichment analysis. Seurat v3 (https://satijalab.org/seurat) was used for scRNA-seq analysis. FKwikQuant Image Analyzer 5.9 (https://kindlebio.com/12-downloads) was used for Western Blot analysis. ImageJ version 1.SIU (https://imagej.nih.gov/ij/) was used for immunofluoresence anlysis. Seahorse XFe 96 Analyzer was used for seahorse analysis (https://www.agilent.com/en/product/cell-analysis/real-time-cell-metabolic-analysis/xf-analyzers/seahorse-xf-pro-analyzer-1980223).

For manuscripts utilizing custom algorithms or software that are central to the research but not yet described in published literature, software must be made available to editors and reviewers. We strongly encourage code deposition in a community repository (e.g. GitHub). See the Nature Portfolio guidelines for submitting code & software for further information.

Data

Policy information about availability of data

All manuscripts must include a data availability statement. This statement should provide the following information, where applicable:

- Accession codes, unique identifiers, or web links for publicly available datasets
- A description of any restrictions on data availability
- For clinical datasets or third party data, please ensure that the statement adheres to our policy

Data sets generated in this study using RNA-seq and scRNA-seq have been deposited at GEO under accession codes GSE192944 (bulk RNA-seq) and GSE196766 (scRNA-seq). The remaining data are available within the Article, Supplementary Information, or Source Data file.
The human genome (hg19) datatbase is accessible at https://www.ncbi.nlm.nih.gov/assembly/GCF_000001405.13/.

Research involving human participants, their data, or biological material

Policy information about studies with human participants or human data. See also policy information about sex, gender (identity/presentation), and sexual orientation and race, ethnicity and racism.

Reporting on sex and gender	Seven bone marrow samples from multiple myeloma patients (Age 63-82; 4 male and 3 female samples) were included in the study as reported in Supplementary Table S3. Sex and gender were not specifically considered in the study design.
Reporting on race, ethnicity, or other socially relevant groupings	Patients' characteristics, including race and ethnicity, are included in Supplementary table S3. Race and ethnicity were not specifically considered in this study.
Population characteristics	Patients' characteristics are included in Supplementary Table S3.<br>We analyzed BM samples from 7 myeloma patients who relapsed after proteasome inhibitor treatment (Ninlaro or Bortezomib).
Recruitment	Relapsed/refractory MM from proteasome inhibitor (PI) based therapy patients were selected for this study.
Ethics oversight	BM aspirates from myeloma patients, who relapsed after proteasome treatment and were referred to Department of Medicine and Surgery, University of Parma, and Department of Lymphoma/Myeloma, University of Texas MD Anderson Cancer Center, were obtained after the approval of the corresponding Institutional Review Boards and in accordance with the Declaration of Helsinki. We do not report any self-selection bias or other biases.

Note that full information on the approval of the study protocol must also be provided in the manuscript.

Field-specific reporting

Please select the one below that is the best fit for your research. If you are not sure, read the appropriate sections before making your selection.

☒ Life sciences ☐ Behavioural & social sciences ☐ Ecological, evolutionary & environmental sciences

For a reference copy of the document with all sections, see nature.com/documents/nr-reporting-summary-flat.pdf

Life sciences study design

All studies must disclose on these points even when the disclosure is negative.

Sample size	In all our human studies, no prior sample-size calculations were possible to predetermine sample size; human samples were used based on availability of fresh or banked samples and selected based on prior therapies (failure to preteasome inhibitors).<br>In mouse studies, we estimated a number of 7 samples per group to be sufficient to identify differences between groups with 80% power, assuming a large effect size. Thus, we generally began experiments with n>=7 per experimental group and in most cases we performed the experiment in duplicate or even in triplicate. In a few cases (as in the setting of transplantation), mice died during the experiment and the number was reduced to 4-6 mice per group. In those cases, we considered the results as valid only if they were consistent with other experiments and/or if differences between experimental groups were clear and statistically significant.<br>In cell line experiments, sample size was not pre-calculated but at least 3 biological replicates were included per condition to determine statistical significance.
Data exclusions	No data were excluded from analysis.
Replication	Every legend includes the number of repeated experiments that were performed.
Randomization	Mice were randomized according to tumor burden assessed by bioluminescence imaging or BM aspirate analysis obtained after 15 days following transplantation. Randomization does not apply to experiments performed in human samples, as these needed to be pre-selected on the basis of the patient therapy as explained above.
Blinding	Experimental data analysis was performed blindly in all in vitro experiments. Blinding does not apply to randomization in mouse

transplantation because, as explained above, the percent of engraftment of human cells had to be known beforehand to exclude failed grafts. Blinding was not possible in human sample selection because only samples from patients who relapsed after PI-based therapy were included

Reporting for specific materials, systems and methods

We require information from authors about some types of materials, experimental systems and methods used in many studies. Here, indicate whether each material, system or method listed is relevant to your study. If you are not sure if a list item applies to your research, read the appropriate section before selecting a response.

Materials & experimental systems

n/a	Involved in the study
☐	☒ Antibodies
☐	☒ Eukaryotic cell lines
☒	☐ Palaeontology and archaeology
☐	☒ Animals and other organisms
☒	☐ Clinical data
☒	☐ Dual use research of concern
☒	☐ Plants

Methods

n/a	Involved in the study
☒	☐ ChIP-seq
☐	☒ Flow cytometry
☒	☐ MRI-based neuroimaging

Antibodies

Antibodies used

Western blot analysis
anti-ILF2/NF45 (Santa Cruz, sc365068,1:500), anti-vinculin (Sigma, V9131,1:2000), anti-phospho-Histone H2AX (Cell Signaling, 2577S,1:500), anti-cleaved caspase 3 (Cell Signaling, 966S,1:500), and anti-Cas9 (Cell Signaling, 14697S,1:1000), in addition to secondary anti-mouse (Kindle Biosciences LLP, R005,1:2000) and anti-rabbit digital antibodies (Kindle Biosciences LLP, R006,1:2000).

Immunofluorescence analysis
anti-phospho-Histone H2AX (Cell Signaling, 2577S,1:200), anti-DNA2 (Invitrogen, PAS-66086,1:200), and anti-TOM20 (Santa Cruz, sc17764,1:200).

Mitochondria and Nuclear Fractionation Western blot
anti-DNA2 (Invitrogen, PAS-8167,1:1000), anti-vinculin (Sigma, V9131,1:2000), anti-COX IV (Cell Signaling, 4850S,1:1000), and anti-Lamin A (Abcam, ab26300,1:1000).

Flow Cytometry analysis
Primary samples:
APC anti-human CD138 (BD, 347207,1:20)
PerCP cy5.5 anti-human CD90 (Biolegend, 328118), 1:20)
Xenograph experiments:
APC/cy7 anti-mouse cd45 (Biolegend, 103116,1:20)

Validation

Some antibodies used in the study were validated in our previous manuscript (i.e., anti-ILF2/NF45, anti-vinculin, anti-phospho-Histone H2AX, anti-Lamin A, and anti-cleaved caspase 3; Marchesini et al. Cancer Cell 2017). Antibodies meet all of the quality control standards defined by manufactures or were validated by other investigators. Validation statements for the commercial antibodies are available on the manufactures websites:
https://www.scbt.com/p/nf45-antibody-h-4
https://www.scbt.com/p/tom20-antibody-f-10
https://www.sigmaaldrich.com/US/en/product/sigma/v9131
https://www.cellsignal.com/products/primary-antibodies/phospho-histone-h2a-x-ser139-antibody/2577
https://www.cellsignal.com/products/primary-antibodies/cleaved-caspase-3-asp175-antibody/9661
https://www.cellsignal.com/products/primary-antibodies/cas9-s-pyogenes-7a9-3a3-mouse-mab/14697
https://www.cellsignal.com/products/primary-antibodies/cox-iv-3e11-rabbit-mab/4850
https://kindlebio.com/products/29-digital-anti-mouse-hrp.html
https://kindlebio.com/products/30-digital-anti-rabbit-hrp.html
https://www.thermofisher.com/antibody/product/DNA2-Antibody-Polyclonal/PA5-66086
https://www.thermofisher.com/antibody/product/DNA2-Antibody-Polyclonal/PA5-68167
https://www.abcam.com/products/primary-antibodies/lamin-a-antibody-ab26300.html
https://www.bdbiosciences.com/en-us/products/reagents/flow-cytometry-reagents/clinical-diagnostics/single-color-antibodies-asr-ivd-ce-ivd/cd138-apc.347207
https://www.biolegend.com/ja-jp/products/apc-cyanine7-anti-mouse-cd45-antibody-2530
https://www.biolegend.com/en-us/search-results/percp-cyanine5-5-anti-human-cd90-thy1-antibody-4515

Eukaryotic cell lines

Policy information about cell lines and Sex and Gender in Research

Cell line source(s)	JJN3 were obtained from DSMZ (ACC 541)<br>KMS11 cells were obtained from JCRB Cell Bank (JCRB1179)<br>MM1R (CRL-2975), H929 (CRL-3580), and RPMI-8226 (CCL-155)cells were obtained by ATCC:<br>https://www.dsmz.de/collection/catalogue/details/culture/ACC-541<br>https://www.atcc.org/products/crl-2975<br>https://www.atcc.org/products/crl-3580<br>https://www.atcc.org/products/ccl-155
Authentication	Cell lines' identity was validated by STR DNA fingerprinting using the Promega 16 High Sensitivity STR Kit. Validations were performed every 6 months.
Mycoplasma contamination	Mycoplasma testing was routinely performed on all cell lines. All cell lines were tested negative for mycoplasma.
Commonly misidentified lines (See ICLAC register)	No misidentified lines were used for the study.

Animals and other research organisms

Policy information about studies involving animals; ARRIVE guidelines recommended for reporting animal research, and Sex and Gender in Research

Laboratory animals	4-week-old BALB-cj (Strain #:0006514) and NSG (Strain #:005557) mice were obtained from the Jackson Laboratory. Mice were maintained under specific-pathogen-free conditions and housed in a barrier facility at 25C under ambient oxygen conditions in a 12-h light/12-h dark cycle under 50% humidity.
Wild animals	The study does not include any wild animals.
Reporting on sex	All transplanted mice were female, which allowed for better engraftment of human cells
Field-collected samples	The study does not include any such samples.
Ethics oversight	All animal experiments were performed with the approval of MD Anderson's Institutional Animal Care and Use Committee.

Note that full information on the approval of the study protocol must also be provided in the manuscript.

Plants

Seed stocks	*Report on the source of all seed stocks or other plant material used. If applicable, state the seed stock centre and catalogue number. If plant specimens were collected from the field, describe the collection location, date and sampling procedures.*
Novel plant genotypes	*Describe the methods by which all novel plant genotypes were produced. This includes those generated by transgenic approaches, gene editing, chemical/radiation-based mutagenesis and hybridization. For transgenic lines, describe the transformation method, the number of independent lines analyzed and the generation upon which experiments were performed. For gene-edited lines, describe the editor used, the endogenous sequence targeted for editing, the targeting guide RNA sequence (if applicable) and how the editor was applied.*
Authentication	*Describe any authentication procedures for each seed stock used or novel genotype generated. Describe any experiments used to assess the effect of a mutation and, where applicable, how potential secondary effects (e.g. second site T-DNA insertions, mosiacism, off-target gene editing) were examined.*

Flow Cytometry

Plots

Confirm that:

- [x] The axis labels state the marker and fluorochrome used (e.g. CD4-FITC).
- [x] The axis scales are clearly visible. Include numbers along axes only for bottom left plot of group (a 'group' is an analysis of identical markers).
- [x] All plots are contour plots with outliers or pseudocolor plots.
- [x] A numerical value for number of cells or percentage (with statistics) is provided.

Methodology

Sample preparation	Single-cell suspensions were prepared from the cell lines or mouse bone marrow samples. Cell clumps were removed using the pre-separation filters (Miltenyi; cat number 130-041-407). Alive cells were counted using trypan blue and further processed for flow cytometry or FACS analysis. Primary bone marrow mononuclear cells isolated from myeloma patients or healthy donors were enriched in CD138+ plasma cells using magnetic sorting with the CD138 Microbead Kit (Miltenyi Biotec). Cells were plated in 48-well plates, previously seeded with human bone marrow-derived mesenchymal cells, and sorted using the CD138 antibody.
Instrument	BD Influx sorter; BD Fortessa analyzer
Software	BD FACSDiva 8.01
Cell population abundance	In preliminary validation experiments in which we re-run sorted samples to evaluate if the purification was successful, the purity of sorted plasma cells was over 95%. Given that in the experiments included in the paper (FACS purification for scRNA-seq or functional assays) we sorted very low numbers of plasma cells (50,000 cells, depending on the experiment), it was impossible to validate each individual experiment again.
Gating strategy	Xenograft experiments:<br>To sort human myeloma cells from the xenografts, the following population was sorted: Live/mCD45-/GFP+ cells.<br><br>Primary samples:<br>To sort primary plasma cells from myeloma patients or healthy donors, the following population was sorted: Live/CD138+ cells.

☒ Tick this box to confirm that a figure exemplifying the gating strategy is provided in the Supplementary Information.
